# PBP2b plays a key role in both peripheral growth and septum positioning in *Lactococcus lactis*

**DOI:** 10.1371/journal.pone.0198014

**Published:** 2018-05-23

**Authors:** Blandine David, Marie-Clémence Duchêne, Gabrielle Laurie Haustenne, Daniel Pérez-Núñez, Marie-Pierre Chapot-Chartier, Xavier De Bolle, Eric Guédon, Pascal Hols, Bernard Hallet

**Affiliations:** 1 Institut des Sciences de la Vie (ISV), Université catholique de Louvain (UCL), Louvain-la-Neuve, Belgium; 2 Micalis Institute, INRA, AgroParisTech, Université Paris-Saclay, Jouy-en-Josas, France; 3 Microorganisms Biology Research Unit (URBM), University of Namur (UNamur), Namur, Belgium; 4 STLO, INRA, Agrocampus Ouest, Rennes, France; Centre National de la Recherche Scientifique, Aix-Marseille Université, FRANCE

## Abstract

*Lactococcus lactis* is an ovoid bacterium that forms filaments during planktonic and biofilm lifestyles by uncoupling cell division from cell elongation. In this work, we investigate the role of the leading peptidoglycan synthase PBP2b that is dedicated to cell elongation in ovococci. We show that the localization of a fluorescent derivative of PBP2b remains associated to the septal region and superimposed with structural changes of FtsZ during both vegetative growth and filamentation indicating that PBP2b remains intimately associated to the division machinery during the whole cell cycle. In addition, we show that PBP2b-negative cells of *L*. *lactis* are not only defective in peripheral growth; they are also affected in septum positioning. This septation defect does not simply result from the absence of the protein in the cell growth machinery since it is also observed when PBP2b-deficient cells are complemented by a catalytically inactive variant of PBP2b. Finally, we show that round cells resulting from β-lactam treatment are not altered in septation, suggesting that shape elongation as such is not a major determinant for selection of the division site. Altogether, we propose that the specific PBP2b transpeptidase activity at the septum plays an important role for tagging future division sites during *L*. *lactis* cell cycle.

## Introduction

Ovococci are ovoid bacteria that exhibit a specific oblong cell morphology. As opposed to “true”, spherical cocci (e.g. *Staphylococcus aureus*), they divide in parallel planes, perpendicularly to their longitudinal axis, which results in the formation of diplococci or cell chains of various lengths [1]. Well-known pathogens belonging to streptococci and enterococci (e.g. *Streptococcus pneumoniae*, *Enterococcus faecalis*) are part of this morphotype, which renders the understanding of their cell cycle of particular importance for the development of new drug-based therapies [2].

As for rod-shaped bacteria (i.e. *Escherichia coli* and *Bacillus subtilis*), the ovoid cell cycle is characterized by two growth phases: peripheral growth, which is responsible for cell elongation, and divisional growth, which leads to cell septation [1–3]. The ovoid cell cycle begins when new cell wall is incorporated at the center of the cell in an outgrowth of accumulated PG called “equator” or “piecrust” [4]. Van-FL or D-amino acids staining identified the septum and the equator as the sites for new cell-wall incorporation in pneumococcal and lactococcal cells [5,6]. New cell wall insertion splits the initial outgrowth into two new outgrowths, which are progressively pushed away to the cell poles and lead to cell elongation and cross-wall synthesis [2,4,7]. During cell elongation, peripheral growth is active and new PG is incorporated at the cell periphery. Afterwards, the septal growth is activated at a specific checkpoint of the cell cycle and new PG is added along the leading edge of the constricting ring [2,6,7].

In rod-shaped bacteria, cell growth is performed by multi-enzymatic machineries specific to elongation or division. These machineries group specific cell wall enzymes such as Penicillin Binding Proteins (PBPs) and peptidoglycan (PG) hydrolases, cytoskeletal proteins, scaffold proteins, and several other proteins of unknown function [8]. The cytoskeletal proteins MreB and FtsZ are key central players in the elongation and division processes, respectively, being the dynamic organizers of the assembly of two multi-enzymatic complexes, called elongasome and divisome [8]. Specific division (FtsZ, ZapA, EzrA, FtsA, DivIVA, FtsE/X, FtsK, DivIB, FtsL, DivIC, and FtsW) and elongation (MreC, MreD, RodA) determinants are found in ovococci but the key peripheral cytoskeleton protein MreB is absent [2]. Their genomes generally encode 6 PBPs: three bifunctional class A PBPs with transglycosylase and transpeptidase activities (PBP1a, PBP1b, PBP2a), two monofunctional class B transpeptidases (PBP2b and PBP2x), and one D,D-carboxypeptidase (DacA) [1,3,9]. The two monofunctional transpeptidases PBP2b and PBP2x are associated to peripheral and divisional cell growth, respectively [2,3,9]. In *S*. *pneumoniae*, PBP2x is essential for growth and the morphological impact of PBP2x depletion was studied through the construction of conditional mutants [5,10,11]. PBP2x-depleted cells exhibited a complex phenotype with swollen, lemon-shaped elongated cells, often with pointed ends [5,10,11]. Interestingly, the β-lactam methicillin was shown to inhibit more specifically PBP2x in *S*. *pneumoniae* and *L*. *lactis* [5,9,12,13]. Consistent with its central role in cell division, inhibition of PBP2x transpeptidase activity led to cell filamentation in both species [5,9,12,13], but with the production of longer filaments in *L*. *lactis* [9,12].

The cell-elongation transpeptidase PBP2b is also essential in *S*. *pneumoniae* [10,14], but not in *L*. *lactis* or *S*. *thermophilus* [9,15]. Depletion of PBP2b in *S*. *pneumoniae* gave rise to long chains of lentil-shaped cells [10], while its inactivation in *L*. *lactis* and *S*. *thermophilus* led to rounded cells [9,15]. These phenotypes are consistent with a role of PBP2b in cell elongation [3,9,10].

In *S*. *pneumoniae*, the current model supposes that ovoid growth results from the activity of a single large machinery for both cell elongation and division at the division site. The composition of the machinery varies in time and space with the MapZ-FtsZ relay acting as a recruiter of machinery compounds for the next growth step [2,16,17]. MapZ (or LocZ) is a recently discovered membrane protein with an extracellular PG binding domain that positively directs FtsZ to the future division site in *S*. *pneumoniae* and probably other ovococci [18,19]. Although all PBPs tent to co-localize in the septal region of *S*. *pneumoniae* [20], PBP2x was recently shown to separate and move toward the inner part of the septum during mid-to-late division stages, while PBP2b, PBP1a and the cell wall regulators MreC and StkP remained at the periphery of the cell [5,13].

*L*. *lactis* is an interesting model for the study of the ovoid cell cycle for the following reasons: (i) division of newborn cells does not take place before division of the mother cell is completed, while overlapping rounds of growth and division are observed in *S*. *pneumoniae* [6], (ii) it displays a strict elongation phase before constriction which is unique among ovococci [6] and (iii) cell elongation and cell division can be uncoupled under defined growth conditions, leading to filamentous cells during planktonic growth as well as in biofilms [9].

Since *L*. *lactis* supports peripheral growth independently of active cell division, we investigate here the specific role of the mono-functional transpeptidase PBP2b during both vegetative and filamentation cell cycles. Notably, we show that the transpeptidase activity of PBP2b is not only required for cell elongation as previously reported, but also for proper septum positioning. Since PBP2b remains intimately associated to the division site during the *L*. *lactis* cell-cycle and since proper ovoid shape itself is not required for septum positioning, we hypothesize that the transpeptidase activity of PBP2b in the septal region may generate a unique PG signature that is required to label the future division site of the cell.

## Materials and methods

### Bacterial strains, plasmids, and growth conditions

The bacterial strains and plasmids used in this study are listed in Table 1. *E*. *coli* was cultivated in Lysogeny Broth (LB) medium [21] at 37°C. The *L*. *lactis* strain NZ3900 is a derivative of the wild-type MG1363 strain in which the two-component NisRK system was introduced to mediate nisin induction of the *nisA* promoter (P_*nisA*_) [22]. All the *L*. *lactis* strains used in this study were derived from NZ3900. *L*. *lactis* was cultivated at 30°C in the rich medium M17 broth (BD biosciences) supplemented with 0.5% of glucose (M17G). When required, antibiotics (Sigma-Aldrich) were added to the media at the following concentrations; erythromycin (250 μg ml^-1^ for *E*. *coli*; 5 μg ml^-1^ for *L*. *lactis*), chloramphenicol (20 μg ml^-1^ for *E*. *coli*; 10 μg ml^-1^ for *L*. *lactis*), and ampicillin (250 μg ml^-1^ for *E*. *coli*). Low concentrations of methicillin (1.0 μg ml^-1^) and amoxicillin (0.1 μg ml^-1^) were used for specific PBP inhibition assays in *L*. *lactis*. These concentrations were chosen based on microcopy observations in order to induce cell filamentation (methicillin) or to generate round cells (amoxicillin) without promoting cell lysis. Nisin A (Sigma-Aldrich) was used at different concentrations to induce expression from P_*nisA*_, depending on the expressed protein; 0.015 ng ml^-1^ for FtsZ-Venus and 0.20 ng ml^-1^ for Venus-PBP2b; and 0.05 and 0.1 ng ml^-1^ for complementation experiments.

**Table 1:** Bacterial strains and plasmids.

Strain or plasmid	Characteristic(s)	Source or reference
Strains		
*L*. *lactis*		
NZ3900	MG1363 derivative containing the *nisR* and *nisK* genes stably integrated at the *pepN* locus	[22]
BLD001	NZ3900 *pbp1a*::pCM1837	This study
BLD002	NZ3900 *pbp1b*::pED 2045	This study
BLD003	NZ3900 *pbp2a*::p2092	This study
BLD004	NZ3900 *pbp2b*::p2081	This study
BLD006	NZ3900 *dacA*::pGIBLD006	[12]
Plasmids		
pCM1837	Em^r^ ^*a*^; suicide plasmid pRV300 containing a ~200-pb disruption cassette of *pbp1a*	[23]
pED2045	Em^r^; suicide plasmid pRV300 containing a 768-pb disruption cassette of *pbp1b*	Domakova E. and Kulakauskas S., INRA ^b^
p2092	Em^r^; suicide plasmid pRV300 containing a 481-pb disruption cassette of *pbp2a*	Budin-Verneuil A. and Maguin E., INRA ^b^
p2081	Em^r^; suicide plasmid pRV300 containing a 1073-pb disruption cassette of *pbp2b*	Budin-Verneuil A. and Maguin E., INRA ^b^
pGIBLD006	Em^r^; suicide plasmid pUC18ery containing a 555-pb disruption cassette of *dacA*	[12]
pNZ8048	Cm^r^ ^*a*^; multicopy plasmid containing the inducible *nisA* promoter (P_*nisA*_), designed for translational fusions	[24]
pGIBLD027	Cm^r^; pNZ8048 derivative containing *pbp2b* under the control of P_*nisA*_	This study
pGIBLD0271	Cm^r^; pNZ8048 derivative containing an inactive variant of *pbp2b* (catalytic residue Ser^414^ of PBP2b mutated in Ala) under the control of P_*nisA*_	This study
pGIBLD008	Cm^r^; pNZ8048 derivative containing the Venus encoding gene.	[25]
pGIBLD031	Cm^r^; pGIBLD008 derivative expressing a FtsZ-Venus fusion protein (Venus in C-terminus of FtsZ).	This study
pGIBLD041	Cm^r^; pGIBLD008 derivative expressing a Venus-PBP2b fusion protein (Venus in N-terminus of PBP2b).	This study

^*a*^ Cm^r^ and Em^r^ indicate resistance to chloramphenicol and erythromycin, respectively.

^*b*^ INRA laboratory collection

### DNA techniques and electrotransformation

General molecular biology techniques were performed according to the instructions given by Sambrook *et al*. [21]. Electrotransformation of *E*. *coli* was performed as described by Dower *et al*. [26]. Electrocompetent *L*. *lactis* cells were prepared as previously described [27]. PCR were performed with Phusion high-fidelity DNA polymerase (Finnzymes) in a GeneAmp PCR system 2400 (Applied Biosystems). The primers used in this study were purchased from Eurogentec and are listed in S1 Table.

### Construction of PBP-deficient strains

PBP mutants of *L*. *lactis* were constructed by simple cross-over. Disruption plasmids carrying an internal fragment of the *pbp* gene to be inactivated (Table 1) were electro-transformed in strain NZ3900 and the resulting mutant strains were selected on erythromycin. The primers used for validation of gene disruption by PCR are listed in the S1 Table. Individual mutants were also validated by PBP profiling (see Results).

### Construction of complementation plasmids

The *pbp2b* coding sequence was amplified from NZ3900 by PCR using the primer pair BlD-PBP2bUpNcoI/BlD-PBP2bDownXbaI (S1 Table). The amplicon was digested with *Nco*I and *Xba*I and then cloned into an *Nco*I/*Xba*I-digested pNZ8048 vector [24]. The resulting multicopy plasmid pGIBLD027 contains the nisin-inducible P_*nisA*_-*pbp2b* expression cassette. The catalytic mutant of PBP2b (Ser^414^ into Ala, named PBP2b*) was obtained by QuickChange Site-Directed Mutagenesis [28] using the primer pair PBP2B*Up/PBP2B*Down and pGIBLD027 as a template (S1 Table). Insertion of the correct mutation in the resulting plasmid pGIBLD0271 was confirmed by DNA sequencing.

### Construction of Venus fusions for protein localization

The *ftsZ* and *pbp2b* coding sequences were amplified from NZ3900 by PCR using the primer pairs MBO-FtsZUp4X4/MBO-FtsZDOWn4X4 and BlD-PBP2bUp4X4/BlD-PBP2bDown4X4, respectively. The *ftsZ* amplicon was restricted by *Nco*I and *Xba*I and cloned into the *Nco*I/*Xba*I-digested pGIBLD008 vector [25]. The resulting multicopy plasmid pGIBLD031 contains the inducible P_*nisA*_-*ftsZ*::*venus* (FtsZ-Ve) expression cassette. The *pbp2b* PCR amplicon was restricted with *Nco*I and *Xba*I and cloned in the *Pci*I/*Spe*I-restricted pGIBLD008 vector. The resulting multicopy plasmid pGIBLD041 carries the inducible P_*nisA*_-*venus*::*pbp2b* (Ve-PBP2b) fusion.

### PBP profiling and β-lactam competition assay

PBP profiling on *L*. *lactis* was inspired by the protocol of Scheffers et al. [29]. *L*. *lactis* cells were cultivated in M17G broth at 30°C, collected during mid-exponential phase, washed in phosphate-buffered saline (PBS, 137 mM NaCl, 2.7 mM KCl, 10 mM Na_2_HPO_4_ and 2 mM KH_2_PO_4_, pH 7.4), and resuspended in buffer A (50 mM Tris-HCl, 50 mM NaCl, 1 mM EDTA, pH 8.0) supplemented with a protease inhibitor cocktail (cOmplete^TM^ EDTA-free, Roche). Cells were disrupted with glass beads using FastPrep [30]. Cell fragments were removed by centrifugation at 20,000 *g* during 5 min. For PBP profiling, membranes were extracted from cell suspensions by differential high-speed centrifugation. The supernatant was collected after two 100,000-*g* centrifugations of 10 and 60 min at 4°C. Pellets were collected and re-suspended in buffer A supplemented with cOmplete^TM^ EDTA-free. Approximately 20 μg of extract were labelled with 10 μM of fluorescent Bocillin (Boc-FL or Boc-650/665; Sigma) during 30 min at 30°C. For the competition experiments, membrane extracts were first incubated with different concentrations of methicillin or amoxicillin during 20 min at 30°C before being labelled with fluorescent Bocillin. Samples were separated on pre-cast 4–20% SDS-PAGE gels (PIERCE). Bocillin-labelled proteins were visualized using an Ettan DIGE fluorescence scanner (GE Healthcare). Gels were then stained with Instant Blue for global protein visualization (Gentaur).

### Detection of Venus fusion proteins

For the Venus-PBP2b fusion (Ve-PBP2b), samples were prepared as described above for PBP profiling. The same sample was used for Venus detection by Western blot and Bocillin binding assay. For the FtsZ-Venus fusion (FtsZ-Ve), cells expressing the fusion protein were grown exponentially and harvested by centrifugation. The pellets were washed twice with PBS and disrupted with glass beads by FastPrep. The cell debris were removed by centrifugation at 20,000 *g* during 5 min. The soluble fraction was collected and incubated with Laemmli buffer at 95°C during 5 min, before separation.

For Western blot analysis, samples were separated on a 4–20% SDS-PAGE pre-cast gel (PIERCE) and proteins were transferred to a Hybond-C Extra Nitrocellulose membrane (Amersham Biosciences). A specific anti-GFP monoclonal antibody (mouse JL8, Living Colors®) was used for Venus detection. A secondary, HRP-conjugated rabbit anti-mouse pAb antibody was used to detect the anti-GFP-Venus complexes. Western blots were revealed by chemiluminescence using the HRP detection kit (NEL104, NEN™ *RENAISSANCE®*). For in gel detection of fluorescent proteins, SDS-containing polyacrylamide gels were washed twice (2 × 15 min) with deionized water to remove SDS. Fluorescent fusion proteins were directly visualized using an Ettan DIGE fluorescence scanner (GE Healthcare).

### Phase-contrast and epifluorescence microscopy

For fluorescent analysis, cells from overnight cultures were inoculated into fresh M17G medium supplemented with appropriate antibiotics. Cells were harvested during exponential growth phase, washed twice, and resuspended in PBS for further analysis. Membrane staining with FM4–64 (Molecular Probes) was performed as previously described [31]. Global PBPs staining was performed by incubating the cells with 75 nM of Boc-650/665 on ice during 15–30 min before observation.

For time-lapse experiments, PBS-washed cells were spotted on a microscopy pad composed of M17G, 2% Noble agar (Becton Dickinson) containing the required antibiotics. After the spot had dried, a coverslip was placed on the inoculated pad and nail varnish was used to seal the mounting. For wild-type, methicillin and/or amoxicillin-treated *L*. *lactis* cells, pictures were taken every 5 min during 150 min. For the FtsZ-Venus fusion, cells were grown in M17G supplemented with Nisin A at a concentration of 0.015 ng ml^-1^ (pre-induction), collected at an OD_600_ of ~0.5, and washed with PBS. Cells were then spotted on a modified agarose pad containing 4-fold diluted M17G in order to avoid background fluorescence. Nisin A was added to the pad at a concentration of 0.5 ng ml^-1^. Pictures were taken every 10 min during 90–120 min. Phase-contrast and fluorescent images were acquired with an Axio Observer Z1 inverted microscope (Carl Zeiss) and an Axiocam MRm col charge-coupled device camera. Image analysis was performed with the AxioVision Rel. 4.8 software (Carl Zeiss), MicrobeTracker [32], or MicrobeJ [33].

### Transmission electron microscopy

For transmission electron microscopy (TEM), cells were grown in rich medium (M17G), collected during exponential growth phase, washed in 0.2 M cacodylate buffer (pH 7.4), and harvested. Bacterial pellets were fixed for 2 h in a buffer containing 2.5% glutaraldehyde and 0.1 M cacodylate (pH 7.4) at 4°C. After fixation, pellets were washed and post-fixed overnight with a 1% osmium tetroxide-containing buffer at 4°C, washed again and dehydrated with increasing percentage of ethanol solutions (30%, 70%, 85% and 99°%). Samples were embedded in epoxy resin, thin-sliced and stained with uranyl acetate and Reynolds lead acetate. Samples were observed with a TEM (Tecnai 10, Philips) at the microscopy department of the University of Namur, Belgium.

## Results

### PBP2b localizes at the division site during most of the vegetative cell-cycle of *L*. *lactis*

**Fig 1:**
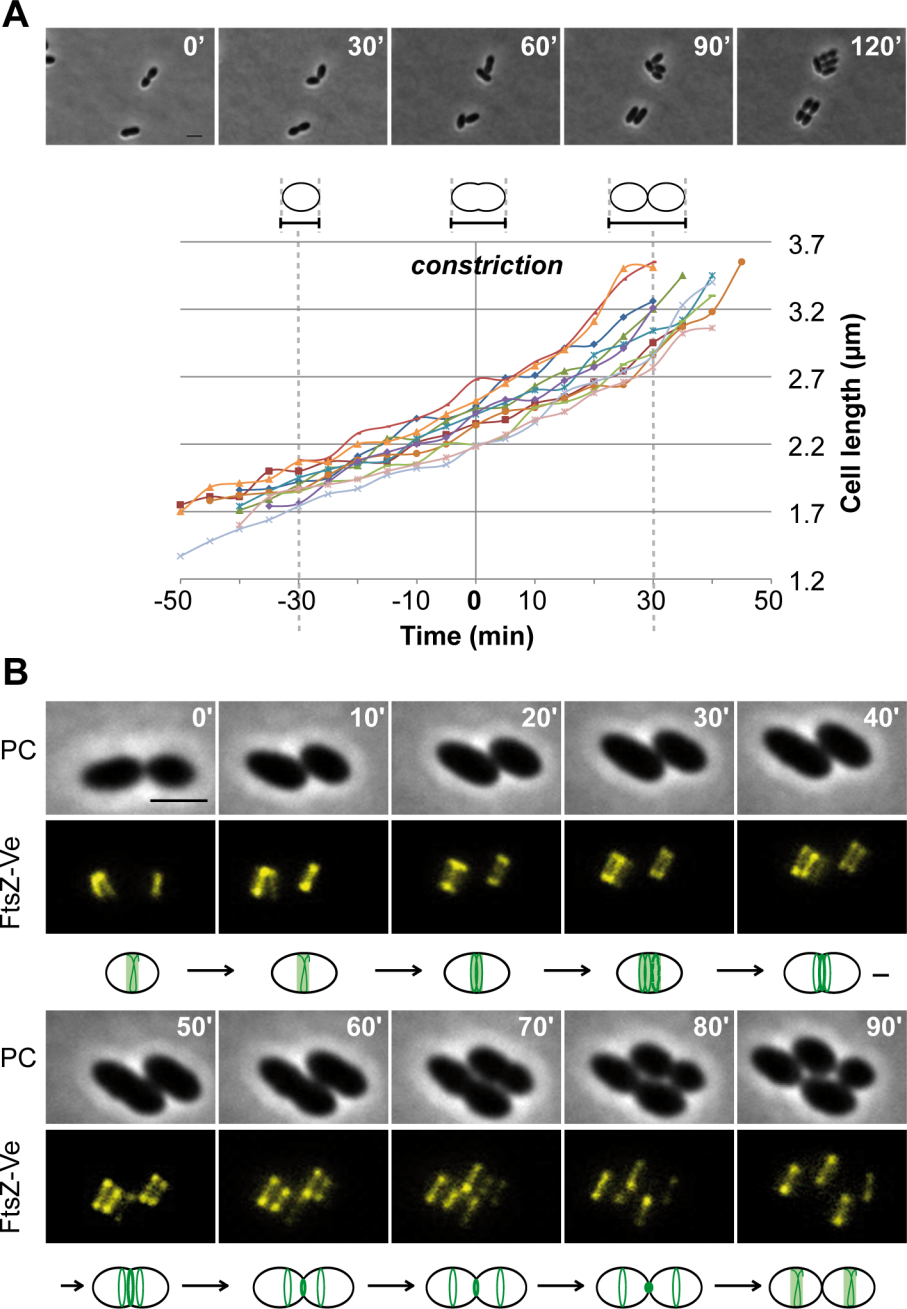
Cell elongation and dynamics of FtsZ during the vegetative cell cycle of *L*. *lactis*. (A) Cell length (in μm) was measured for 11 individual cells analyzed by time-lapse microscopy (representative example shown on the top) and the resulting length curves were aligned on the start of cell constriction (T_0_ = 0 min). The cell cycle is separated in two phases without obvious transition between: i. cell elongation only and ii. combined elongation and division during and after constriction. (B) Time-lapse imaging of FtsZ-Ve during cell elongation and division. *L*. *lactis* cells expressing the FtsZ-Ve fluorescent protein (NZ3900 [pGIBLD031]) were grown on agar pads and visualized by phase contrast (PC, top row) and epifluorescence (FtsZ-Ve, lower row) microscopy (see also S3 Fig [Cell #1] and S1 and S2 Movies). Pictures were taken every 10 min. Observed structural changes of the FtsZ ring (green line) are schematically represented for different steps of the cell cycle. The shaded green band depicts the fuzzy aspect of FtsZ structures during early elongation phase. Scale bar, 2μm.

As the cell cycle of *L*. *lactis* displays unique features among ovococci (see introduction), the subcellular localization of PBP2b during the vegetative cell cycle was examined. As a preliminary step, we first reinvestigated the cell cycle using time-lapse experiments. We measured the elongation rate of individual cells before and after cell constriction. In agreement with previously published experiments [6], *L*. *lactis* displays a strict elongation phase (length increase of ~ 0.6–0.7 **μ**m) before constriction followed by a combined division/elongation phase with an additional cell length increase of 0.6–0.7 **μ**m (Fig 1A). In some rod-shaped Gram-positive bacteria, it has been reported that cells stop to elongate when they start dividing and reciprocally [25,34,35]. During *L*. *lactis* cell cycle, no strict transition between both phases is observed.

In ovococci, FtsZ is the only identified component of the cytoskeleton that could orchestrate both cell elongation and cell division [2,36]. To visualize the position of FtsZ during the cell cycle, we expressed a fusion between *L*. *lactis* FtsZ and the yellow fluorescent protein ‘Venus’ (FtsZ-Ve, C-terminal fusion, S1 Fig) from a multicopy plasmid (strain NZ3900 (WT) containing plasmid pGIBLD031). Examination of the subcellular localization of FtsZ-Ve by time-lapse imaging clearly identified separate phases during the cell cycle of *L*. *lactis* (Fig 1B, S2 and S3 Figs, and S1–S4 Movies). At the onset of the cycle, FtsZ forms a discrete ring at mid-cell. Early during elongation, this initial ring re-organizes to form a doublet or a distorted ‘V-like’ structure at the level of the septal zone (Fig 1B, S2 and S3 Figs). Double rings or ‘V-like’ structures were equally observed during early elongation (*n* = 42 cells from 3 independent time-lapse experiments). In few cells (< 5%), interconversion between ‘V-like’ structures and doublets could be visualized (Fig 1B and S3 Fig), suggesting a dynamic process. When constriction begins, this structure segregates into three components; the actively constricting Z-ring at the middle of the cell, and two lateral rings that progressively migrate towards the center of the future daughter cells. After complete constriction of the median Z-ring, these new lateral rings position FtsZ for triggering a new cycle in the newborn cells (Fig 1B). This phase of concomitant cell elongation/division and the absence of division in daughter cells before completion of the division of the mother cell were systematically observed (*n* = 22 cells from 3 independent time-lapse experiments).

**Fig 2:**
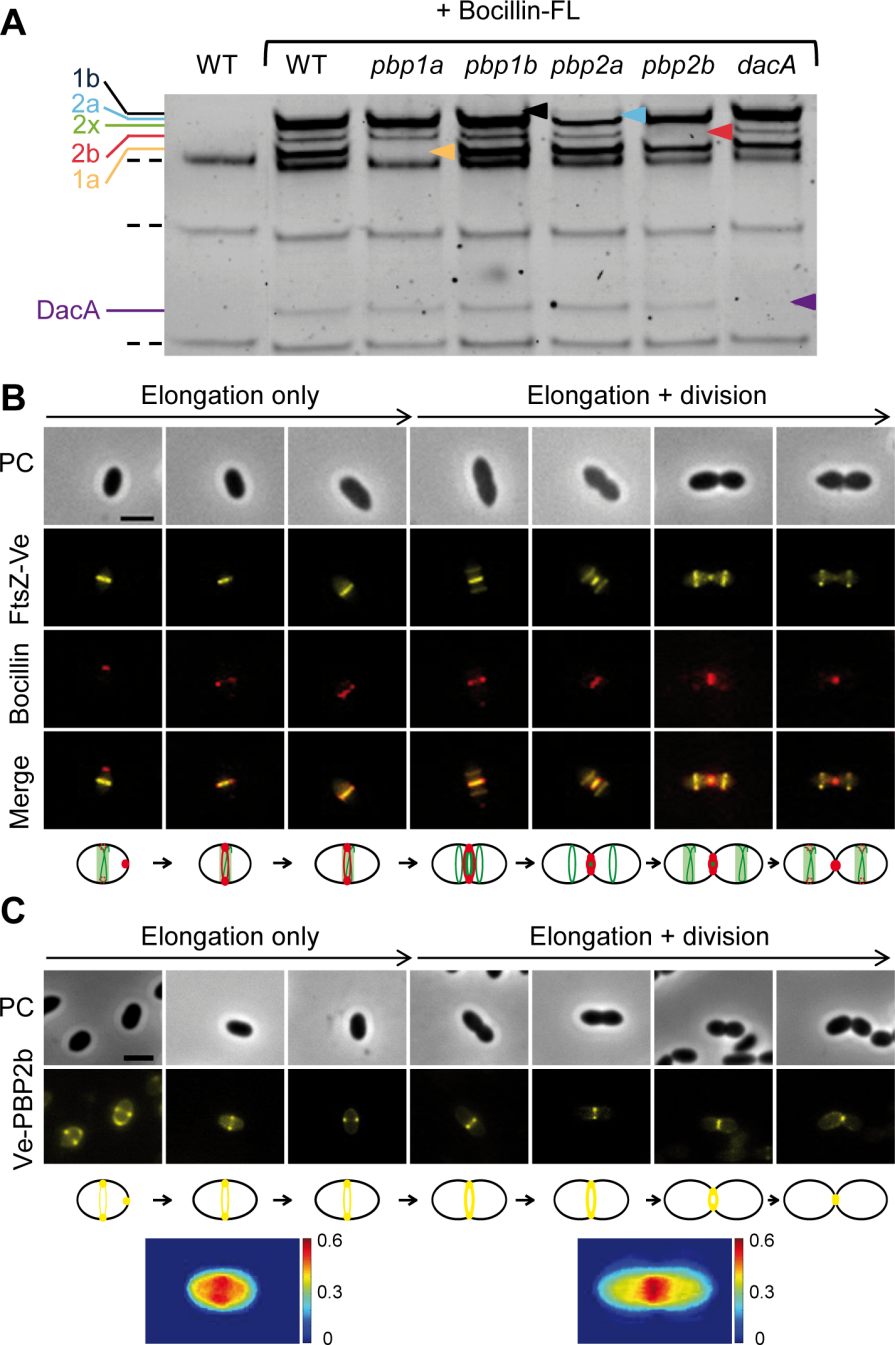
Localization of PBPs and PBP2b during the vegetative cell cycle of *L*. *lactis*. (A) Labelling of *L*. *lactis* PBPs by Bocillin-FL staining. Membranes of wild type (WT), *pbp1a*, *pbp1b*, *pbp2a*, *pbp2* and *dacA* mutant cells were purified, incubated with Bocillin-FL (+ Bocillin-FL), and separated on SDS polyacrylamide gel. Bocillin-FL-labeled PBP bands were revealed by fluorescence scanning. Dotted lines indicate auto-fluorescent bands detected in wild-type extracts prior to Bocillin-FL staining. Colored arrowheads mark the absence of PBP1b (1b, black), PBP2a (2a, light blue), PBP2b (2b, red), PBP1a (1a, yellow) and DacA (purple) in the mutant profiles, except for PBP2x whose deleted mutant is not viable. (B) Localization of PBPs with respect to FtsZ during the cell cycle. Cells expressing FtsZ-Ve (NZ3900 [pGIBLD031]) were stained with Bocillin™650/665 and visualized by phase contrast (PC) and epifluorescence (FtsZ-Ve and Bocillin) microscopy. Merge shows the superimposition of both fluorescent patterns. Scale bar, 2μm. *L*. *lactis* cell cycle was reconstituted from individual cells taking the beginning of cell constriction (as visualized by phase contrast) as the demarcation between elongation-only and combined elongation + division. PBP staining by Bocillin™650/665 is depicted in red on the cell cycle diagram shown below the pictures. Scale bar, 2μm. (C) Cells expressing the Venus-PBP2b fusion (Ve-PBP2b) (NZ3900 [pGIBLD041]) were visualized by phase contrast (PC) and epifluorescence microscopy. Scale bar, 2μm. A complete cell cycle was reconstituted from representative cells as reported in panel B. Ve-PBP2b fluorescence pattern is depicted in yellow on the cell cycle diagram shown below the pictures. The two bottom panels show fluorescence intensity maps (in arbitrary units, A.U.) from low (blue) to high (red) intensity). Cells (*n* = 20) were chosen before and after cell constriction based on phase contrast imaging and their normalized Ve-PBP2b fluorescent profiles were superimposed.

In order to correlate FtsZ dynamics with PG synthesis, global localization of the PG biosynthetic enzymes was determined by staining the cells with Bocillin™650/665, a fluorescently-labeled penicillin derivative that labels all 6 PBPs of *L*. *lactis* (PBP1a, PBP1b, PBP2a, PBP2b, PBP2x and DacA) (Fig 2A). Since Bocillin™650/665 treatment affected growth, a complete cell cycle was reconstituted from individual cells based on the time lapse pattern of FtsZ (Fig 2B). Superimposition of FtsZ-Ve and Bocillin™650/665 fluorescence revealed that PBPs re-location exhibits a certain delay with respect to FtsZ during the cell cycle, as was previously reported for *S*. *pneumoniae* [5,20]. In addition, PBP positioning was found to be highly selective with respect to FtsZ structures. In newborn cells, PBPs essentially localized at the new pole prior to migrate to mid-cell where they co-localized with median FtsZ structures for the rest of the cell cycle. The bulk of PBPs appeared to stay at the division site until after completion of septation without joining the new pair of lateral FtsZ rings that formed earlier during the division process (Fig 2B).

Non-discriminating PBPs labeling with Bocillin™650/665 suggests that both septal and peripheral PG synthesis of *L*. *lactis* take place in the same cell area associated with the equatorial ring. In order to distinguish PBP2b localization, a N-terminal fusion between PBP2b and Venus was constructed (Ve-PBP2b). The Ve-PBP2b was expressed in wild-type cells from a multicopy plasmid (strain NZ3900 (WT) carrying plasmid pGIBLD041). The fusion protein was produced as a full-length and active form as determined by western blot, Bocillin-FL staining, and complementation (S1 Fig). Subcellular localization of Ve-PBP2b is reminiscent to the overall PBP staining pattern given by Bocillin™650/665 (compare Fig 2B and 2C). The fusion protein shows a bright and discrete fluorescent signal that remains tightly associated with the equatorial zone and the constricting septum for most of the cell cycle (Fig 2C and S4 Fig). Transient polar localization of Ve-PBP2b is also observed in newborn cells, presumably as a remnant of late PG synthesis occurring during septum closure (Fig 2C and S4 Fig).

Similarly to *S*. *pneumoniae*, these data are consistent with the view that the cytoskeleton protein FtsZ plays an architectural role for the recruitment and positioning of PBPs all over the cell cycle of *L*. *lactis*. Thus, although *L*. *lactis* is peculiar among ovococci by showing a strict elongation phase before constriction, the positioning of all PBPs including the cell elongation PBP2b is restricted to the equatorial zone during most of the cell cycle.

### PBP2b localizes at future division sites during *L*. *lactis* filamentation

**Fig 3:**
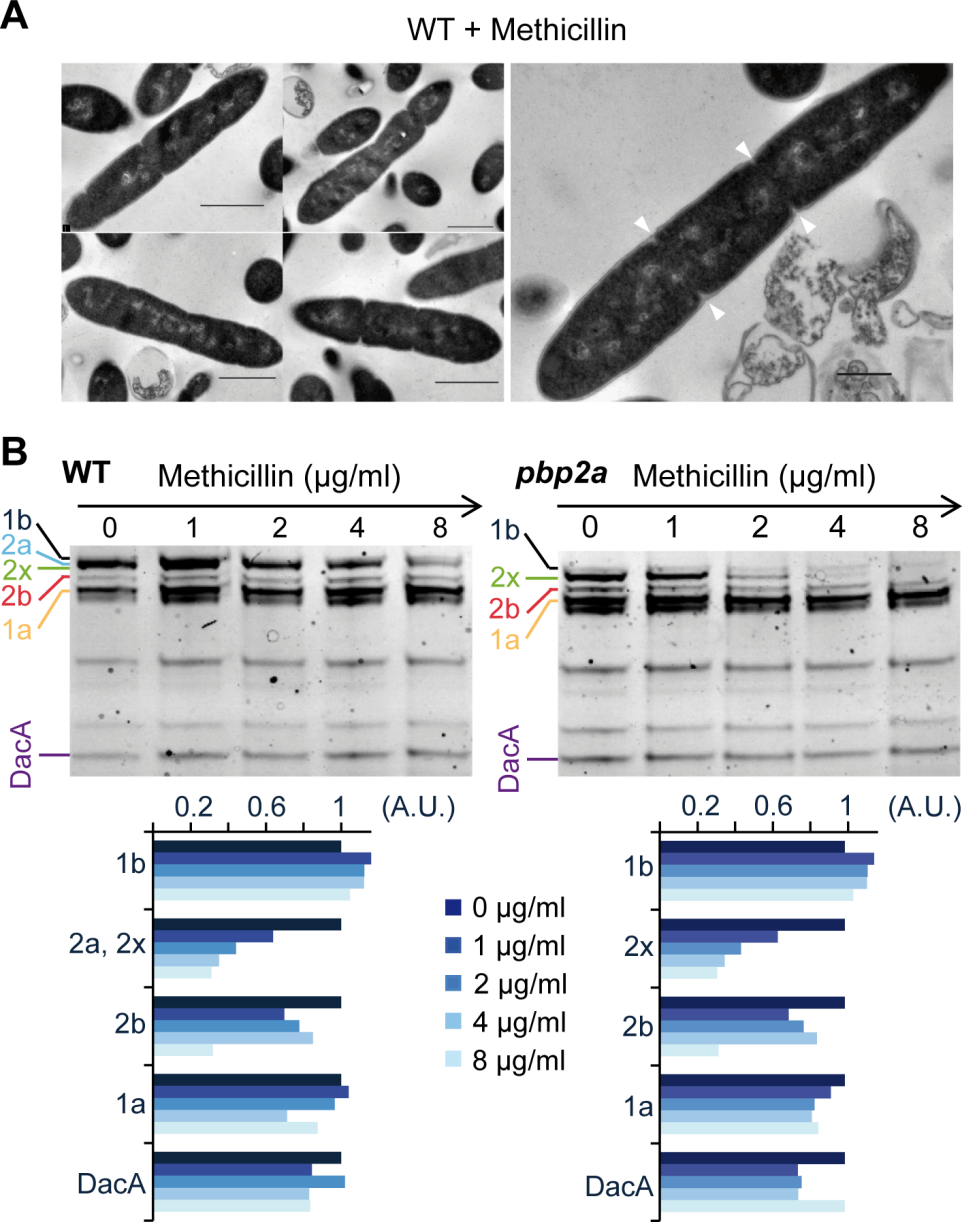
Filamentation of *L*. *lactis* induced by methicillin treatment. (A) Micrographs of wild-type (NZ3900) cells treated by methicillin (1 μg ml^-1^) obtained by transmission electron microscopy (TEM). Arrows indicate incomplete septa. Scale bars, 500 nm. (B) Identification of methicillin-targeted PBPs by Bocillin-FL staining competition assay. Membrane extracts from wild-type and *pbp2a* mutant cells were incubated with 0, 1, 2, 4 or 8 μg ml^-1^ of methicillin prior to add Bocillin-FL. Note the sharp decrease in PBP2x band intensity in the profile of the *pbp2a* mutant. In wild-type extracts, selective blocking of PBP2x by methicillin is masked by the co-migrating PBP2a band. The two bottom panels show the relative fluorescence intensity of each band (in arbitrary units, A.U.) normalized to the fluorescence intensity measured in absence of methicillin (first lane of each gel).

We previously reported that *L*. *lactis* strain IL1403 has the capability to form filaments by septation inhibition during growth in synthetic medium [9]. We have also shown that incubation of *L*. *lactis* IL1403 and MG1363 cells with a low concentration of the β-lactam antibiotic methicillin (i.e. 1.0 μg ml^-1^) induces a filamentation phenotype that we have proposed to result from a specific inhibition of cell division [9]. The morphology of methicillin-treated cells was investigated here by TEM (Fig 3A). As observed in *L*. *lactis* filaments obtained in native conditions [9], methicillin-induced filaments display regular and symmetrical invaginations, interpreted as incomplete septa (Fig 3A). PBP2x is thought to be the primary target for methicillin since mutations conferring methicillin resistance selectively affect the *pbp2x* gene in *L*. *lactis* [9] and other ovococci [37,38]. To confirm this directly in *L*. *lactis*, we performed a competition experiment in which membrane extracts were pre-incubated with increasing amounts of methicillin prior to label PBPs with Bocillin-FL (Fig 3B). In wild-type extracts, selective blocking of PBP2x by methicillin is masked by the co-migrating PBP2a band (Fig 3B, left panel). For this reason, membrane extracts of the *pbp2a* mutant were processed in parallel (Fig 3B, right panel). The resulting PBP profile and its quantification showed a more selective *in vitro* inhibition of the intensity of the band corresponding to PBP2x compared to other Bocillin-FL-labeled bands in a narrow range of methicillin concentrations (2 and 4 μg ml^-1^) (Fig 3B). Altogether, these results support the conclusion that methicillin-induced filamentation results from impairment of septation due to a specific inhibition of PBP2x, which represents an interesting mimetic model for natural filamentation in *L*. *lactis*. Furthermore, it provides an experimental setup for uncoupling cell elongation from cell division.

**Fig 4:**
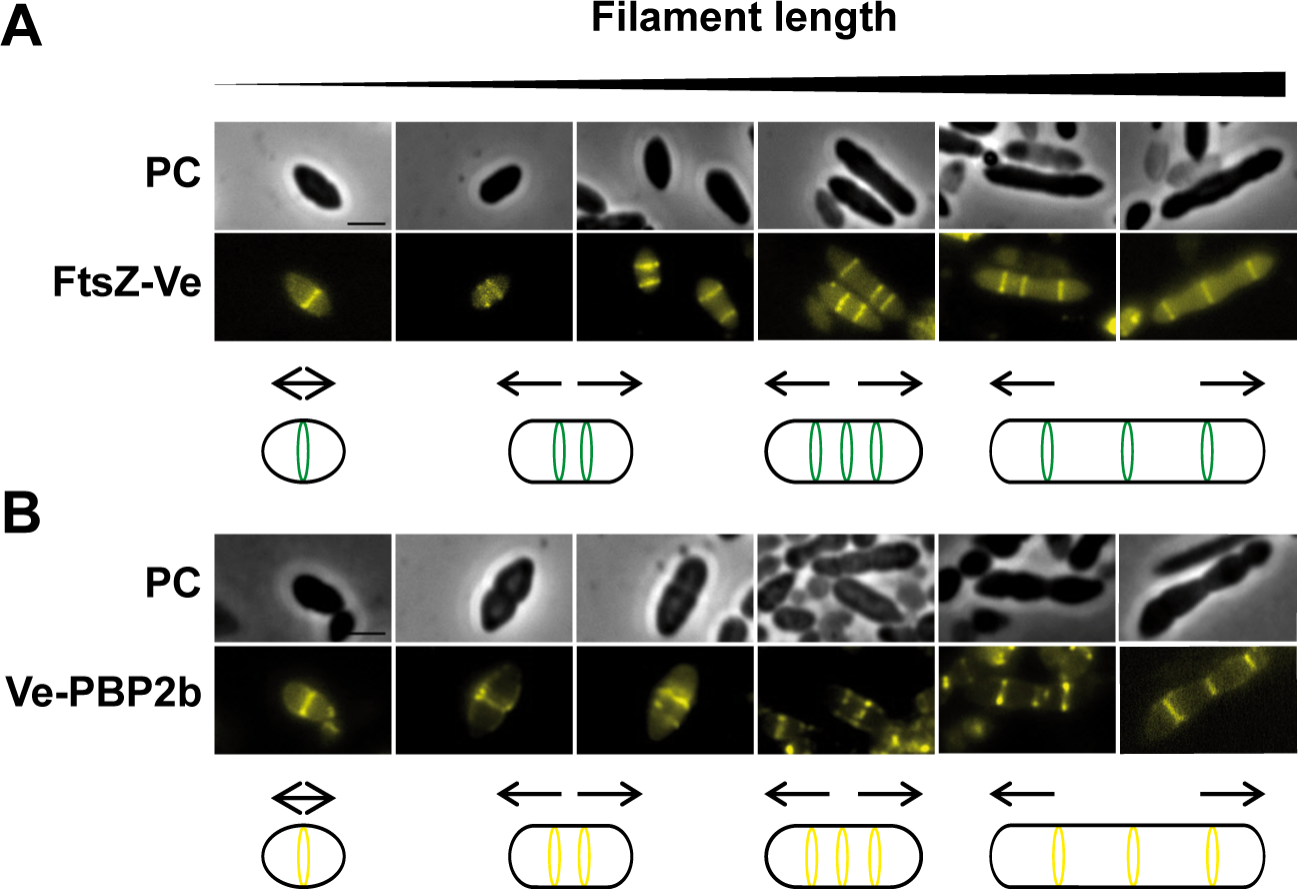
Localization of FtsZ and Pbp2b during filament formation. *L*. *lactis* cells expressing FtsZ-Ve (NZ3900 [pGIBLD031]) (A) or Ve-PBP2b (NZ3900 [pGIBLD041]) (B) were grown in the presence of methicillin 1 μg ml^-1^ to induce filament formation and visualized by phase contrast (PC) and fluorescence microscopy to examine the subcellular localization of the corresponding proteins at different stages of filament elongation. Schematic diagrams summarizing the data are shown below the microscopy pictures. The localization pattern of FtsZ-Ve and Ve-PBP2b are shown in green and yellow, respectively. Scale bars, 2 μm.

To investigate PBP2b dynamics under strict elongation conditions, cells expressing the Ve-PBP2b fluorescent fusion were treated with methicillin (1 μg ml^-1^) and localization of the protein was examined with respect to fluorescent FtsZ-Ve at different stages of filament formation (Fig 4). During filament elongation, FtsZ-Ve showed a typical banding pattern that is consistent with the formation of FtsZ ring-like structures as described above for the vegetative cell cycle (see Fig 1B). The number of rings globally increased with filament length but remained limited to a maximum of 3 to 5 fluorescent bands for the longer filaments (> 5 μm) (Fig 4A and S5A Fig). Expression of Ve-PBP2b in methicillin-treated *L*. *Lactis* cells resulted in the formation of ‘lumpy’ filaments, presumably due to some disturbance of the expressed protein (Fig 4B). Nevertheless, the cellular pattern of Ve-PBP2b appeared to match the pattern of FtsZ-Ve during the filamentation process (Fig 4). As observed for FtsZ ring-like structures, the number of fluorescent bands increased with filament length and remained limited to a maximum of 3 to 5 (S5B Fig). In addition, the bulk of PBPs (Bocillin™650/665 labeling) essentially co-localized with FtsZ ring-like structures in filaments as observed during the vegetative cell cycle (S6 Fig).

These results are consistent with the view that in filaments, FtsZ directs sustained peripheral PG synthesis by PBP2b as it does throughout the vegetative cell cycle.

### PBP2b and FtsZ display similar dynamics during reversion of *L*. *lactis* filaments

**Fig 5:**
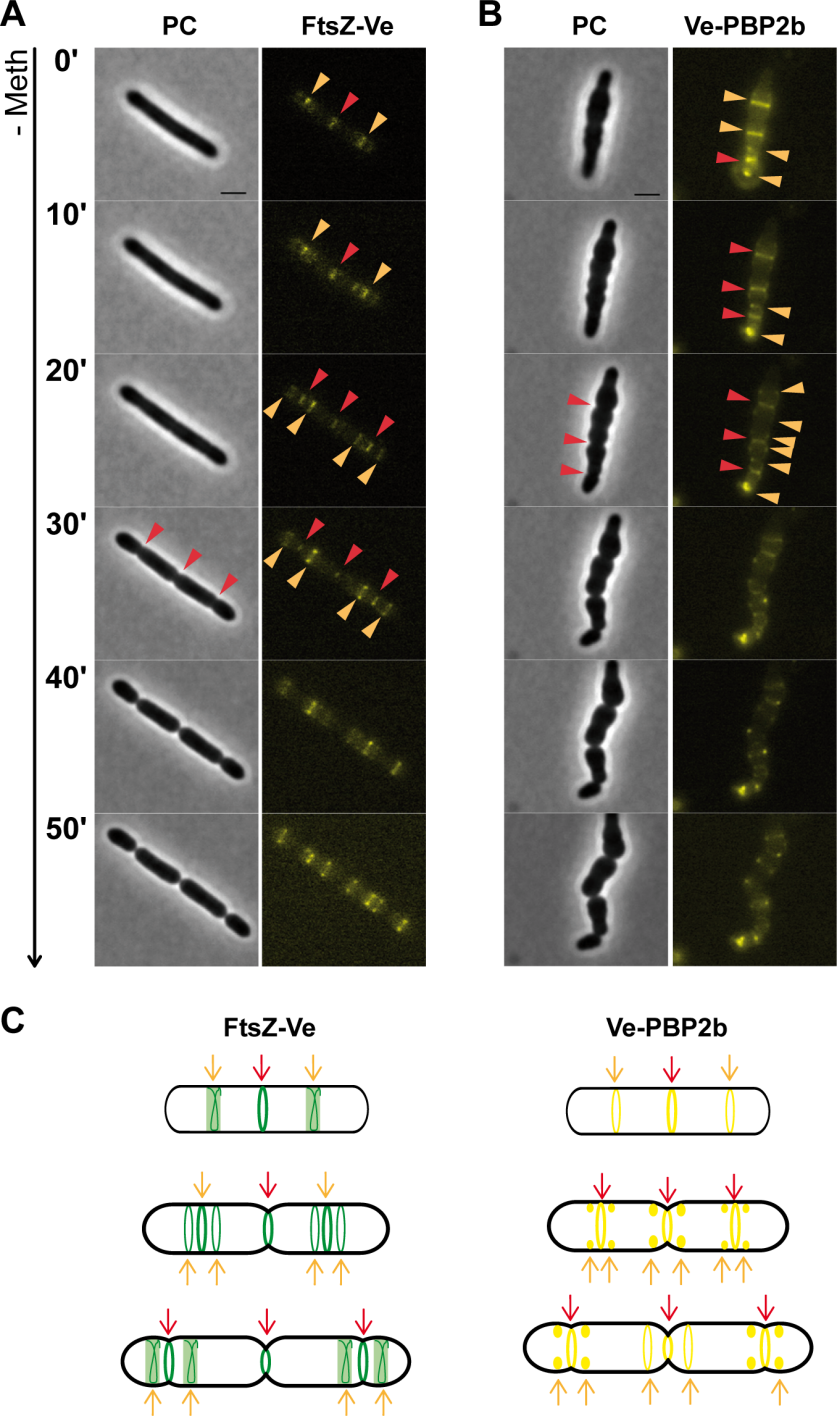
Dynamics of FtsZ and Pbp2b during filament reversion. Methicillin-induced filaments expressing FtsZ-Ve (NZ3900 [pGIBLD031]) (A) or Ve-PBP2b (NZ3900 [pGIBLD031]) (B) were transferred to methicillin-free agar pads to study their localization dynamics by time-lapse microscopy (phase contrast (PC) and fluorescence) during filament reversion (see also S5–S10 Movies). Localization of the protein at the constricting septum and future division sites is highlighted with red and orange arrows, respectively. Only the early steps of filament reversion are highlighted. Scale bars, 2 μm. (C) Schematic diagrams summarizing localization patterns of FtsZ-Ve (green) and Ve-PBP2b (yellow).

An intriguing feature of the methicillin-induced filamentation phenotype lies in its reversibility (Fig 5) [9]. Filaments that were generated by incubating exponentially-growing *L*. *lactis* cells with methicillin were shown to undergo multiple rounds of cell division immediately after being transferred into fresh medium without antibiotics. Time-lapse analysis of the reversion process showed that successive cycles of division occurred in a processive manner, which progressively resolved the filaments into individual cells of normal size (Fig 5, see also S5 and S7 Movies). Intriguingly, each division step was accompanied by a net increase in the length of filaments that corresponded to the elongation rate of non-filamentous cells grown under the same conditions (~1.6 ± 0.2 μm per cell division). This shows that suppressing PBP2x inhibition by removing methicillin from the growth medium does not just reactivate cell septation; it restores the normal growth cycle of *L*. *lactis*.

Localization of Ve-PBP2b was examined with respect to FtsZ-Ve in time-lapse experiments performed during filament reversion (illustrated here for a filament with 3 rings at Fig 5). In the example shown in Fig 5A, the median Z-ring constricted first, immediately followed by the laterally positioned rings (see also S7 and S8 Figs, and S5–S8 Movies). Constriction of the laterally positioned rings was accompanied with the production of two secondary FtsZ rings symmetrically positioned on either side of the constricting ring (Fig 5A, S7 and S8 Figs, and S5–S8 Movies). The same process repeated stepwise until complete reduction of the filaments (S5–S8 Movies). Thus, time-lapse analysis of the reversion process reveals that resolution of methicillin-induced filaments takes place through successive cycles of local growth and division, which are orchestrated by sequential rearrangements of FtsZ structures as it happens during normal growth. Although less clear due to misshaped filaments, the cellular pattern of Ve-PBP2b seemed to match the dynamics of FtsZ-Ve re-localization during filament reversion as was observed during normal division cycles. Ve-PBP2b first formed discrete fluorescent bands that segregated into secondary parietal foci or lateral bands at each new round of cell division (Fig 5B, S8 Fig, and S9 and S10 Movies).

We propose here that FtsZ drives PBP2b during the reversion of filaments to assist consecutive elongation-division phases as it does during normal division cycles.

### PBP2b is required for both cell elongation and proper septation in *L*. *lactis*

**Fig 6:**
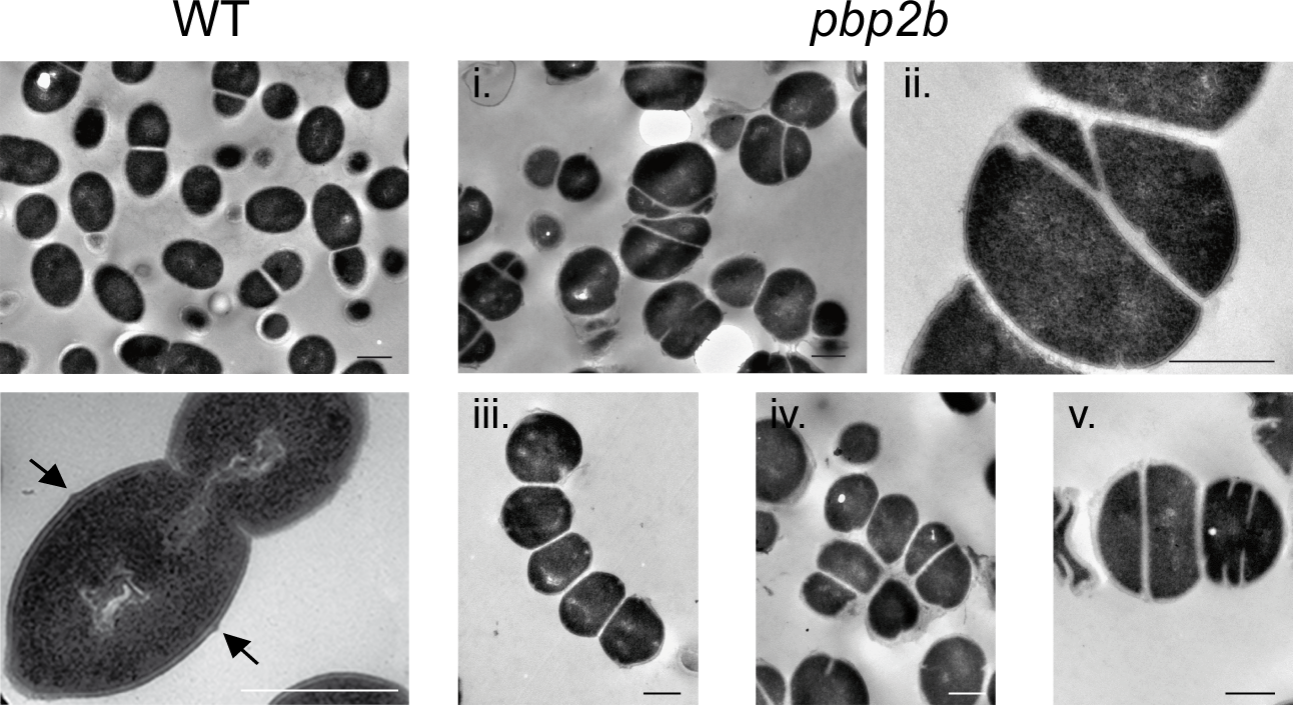
TEM Micrographs of wild-type (WT, NZ3900) and *pbp2b* mutant cells of *L*. *lactis*). (i and ii), *pbp2b* mutant cells with mis-oriented and asymmetrical septa; (iii), small chains of round cells; (iv), aggregate of unseparated cells; (v), cell with double septa. Arrows indicate PG outgrowths (piecrust) at the future septation site in WT. Scale bars, 500 nm.

We previously reported the viability of a mutant deficient for the cell-elongation transpeptidase PBP2b [9]. This distinguishes *L*. *lactis* from the related ovoid bacterium *S*. *pneumoniae* where PBP2b was found to be essential [14]. Still, the *L*. *lactis pbp2b* mutant exhibits a marked growth defect compared to the wild-type (WT) strain (S9 Fig). To investigate the impact of PBP2b deficiency on cell morphology, the mutant was first examined by TEM analysis (Fig 6). Micrographs showed that PBP2b-deficient cells are rounder than WT *L*. *lactis* cells as expected for an impairment in cell elongation (Fig 6). However, their morphology is much more heterogeneous than observed for the depletion of PBP2b in *S*. *pneumoniae*, which essentially gave chains of compressed cells [10]. Although small chains of lentil-shaped cells were observed with the *L*. *lactis pbp2b* mutant, most PBP2b-deficient cells showed a puffy phenotype with mis-positioned and mis-oriented septa, which is indicative of a defect in cell polarity and proper division site positioning (Fig 6). In some cases, aberrant septa appeared to divide the cells into unequal compartments, which is likely to have deleterious consequences on chromosome segregation and cell viability (Fig 6).

**Fig 7:**
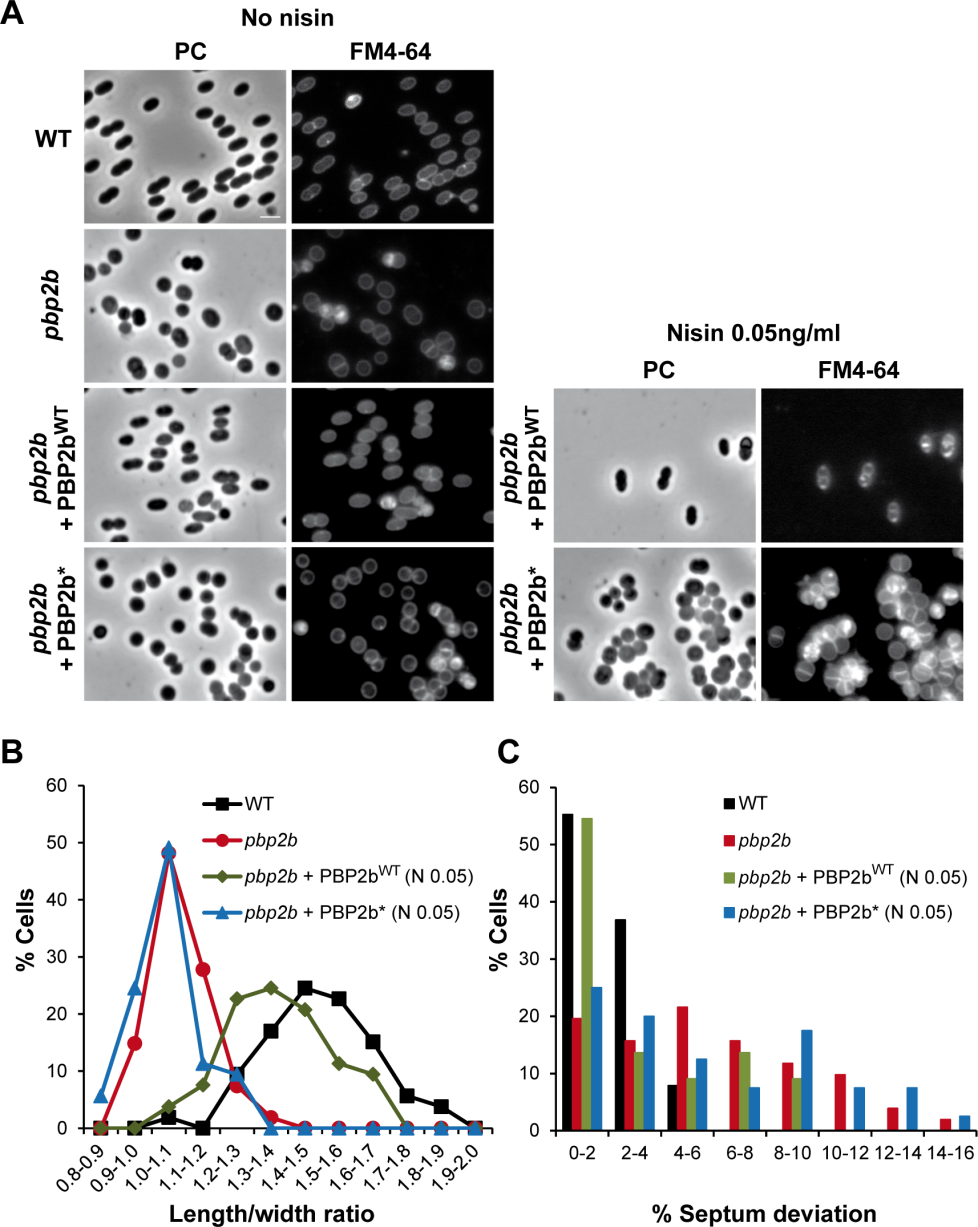
Complementation of *pbp2b* mutant with PBP2b wild type (PBP2b^WT^) and the catalytic mutant of PBP2b (PBP2b*). (A) Images of wild-type (WT, NZ3900), *pbp2b* mutant, *pbp2b* + PBP2b^WT^, and *pbp2b* + PBP2b* cells obtained by phase contrast (PC) and epifluorescence (membrane staining with FM4-64) microscopy. Cells were grown without (no nisin) or with nisin (0.05 ng ml^-1^) as inducer of the expression of *pbp2b* gene variants. (B) Distribution of length to width ratios in the cell population (*n* = ~50) of WT, *pbp2b* mutant, *pbp2b* + PBP2b^WT^ and *pbp2b* + PBP2b*. For complementation, measurements were performed from cells grown with nisin 0.05 ng ml^-1^ (N 0.05). (C) Relative septum position in dividing cells (% of deviation from the median position of cells stained with FM4-64) of the cell population of WT, *pbp2b* mutant, *pbp2b* + PBP2b^WT^ and *pbp2b* + PBP2b* (in presence of nisin 0.05 ng ml^-1^, N 0.05).

The morphological defects of the *pbp2b* mutant were confirmed on live cells by standard phase contrast and epifluorescence microscopy (Fig 7). Mutant cells had a significantly lower length to width (l/w) ratio than WT cells (l/w of 1.07 ± 0.15 *versus* 1.37 ± 0.17, *t* test, *P* < 0.01, *n* = ~50), consistent with their spherical morphology (Fig 7A and 7B). As an approximation of septation defects, we examined the relative septum position of dividing cells (expressed in% of deviation from the median position) from images obtained by epifluorescence microscopy after membrane staining with FM4-64. Mutant cells exhibited much more frequent asymmetrical divisions resulting from misplaced septa than the WT (Fig 7C). Remarkably, similar morphological defects were previously reported for D,D-carboxypeptidase-deficient strains of *L*. *lactis* and *S*. *pneumoniae* [12,39–41].

To rule out the possibility that suppressor mutations or polar effects could interfere with the phenotype, the mutant was complemented by an extra-chromosomal copy of *pbp2b* under the control of the nisin-inducible promoter P_*nisA*_ (Fig 7, *pbp2b* + PBP2b^WT^). In the absence of nisin (low expression due to promoter leakage) or in the presence of a very low amount of the inducer (0.05 ng ml^-1^), most complemented cells recovered their wild-type shape and a correct septum position (Fig 7).

Finally, the *pbp2b* mutant was grown in the absence of erythromycin to allow excision of the disruption plasmid from the *pbp2b* locus. The cell morphology of reverted erythromycin-sensitive clones could not be distinguished from the WT as attested by measurements of cell length and width (S10 Fig), confirming the absence of secondary mutations that could alter cell morphology in the *pbp2b* mutant.

Thus, PBP2b is not only required for peripheral growth but also for the correct positioning of the septum in *L*. *lactis*.

### Proper septation in *L*. *lactis* requires PBP2b transpeptidase activity

To determine whether the physical presence of PBP2b is sufficient to ensure proper septation, or whether it requires the transpeptidase activity of the PG synthase, a catalytically inactive variant of PBP2b (PBP2b*) was constructed by replacing the conserved active site residue Ser^414^ [42] by an alanine. As for WT PBP2b, complementation of the *pbp2b* mutant with PBP2b* (*pbp2b* + PBP2b*) was performed in the absence or presence of low amounts of the nisin inducer (i.e., 0.05 ng ml^-1^; Fig 7). In both cases, the length to width ratio of the cell population remained globally unchanged compared to the *pbp2b* mutant (Fig 7B). In addition, contrarily to PBP2b^WT^, PBP2b* complementation was unable to restore correct positioning of the septum (Fig 6C). From two-fold to ten-fold higher nisin concentration (0.1 to 1 ng ml^-1^), the whole cell population remained spherical with around 50% of dividing cells displaying defects in septum positioning (S11 Fig and data not shown). However, at these higher nisin concentrations, part of the cell population forms chains, which is indicative of an additional defect in cell separation (S11 Fig and data not shown). This suggests that the overproduction of an inactive version of PBP2b disturbs the functioning of PG hydrolases involved in the cell separation process in *L*. *lactis*.

These results show that the transpeptidase activity of PBP2b is essential for cell elongation and that it contributes to proper septum formation either directly or indirectly.

### β-lactam inhibition of cell elongation does not alter proper septation in *L*. *lactis*

**Fig 8:**
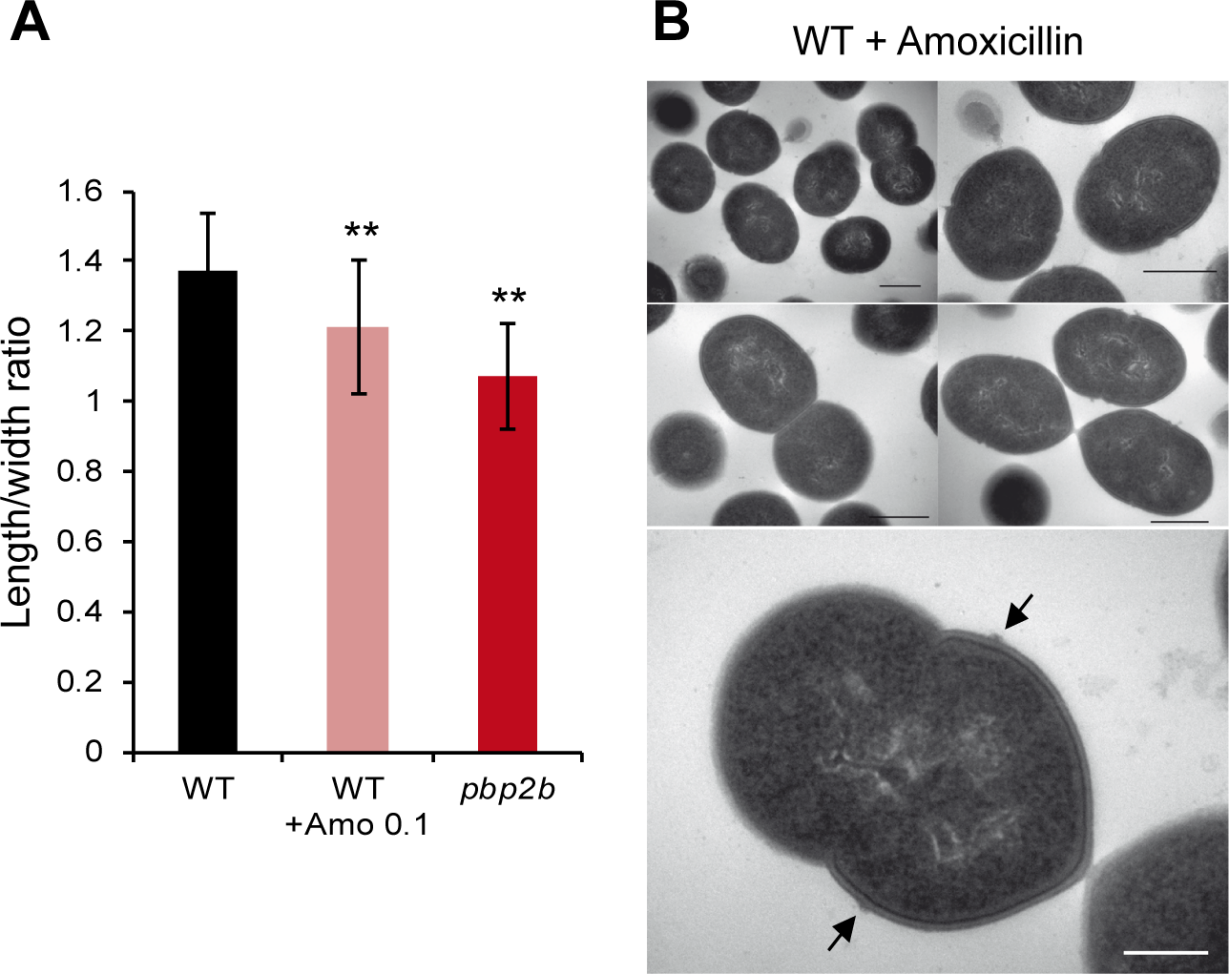
Inhibition of cell elongation in *L*. *lactis* by amoxicillin treatment. (A) Length to width ratios of wild-type (WT, NZ3900), WT treated with amoxicillin 0.1 μg ml^-1^ (WT + Amo 0.1), and *pbp2b* mutant. Mean values (*n* = ~ 50 cells) ± standard deviations. Statistical analysis of the difference between length to width ratios was performed by a *t* test using WT as reference. **, *P* < 0.01. (B) Micrographs of WT cells treated by amoxicillin (0.1 μg ml^-1^) obtained by transmission electron microscopy (TEM). Arrows indicate PG outgrowths (piecrust) at the future septation site. Scale bars, 500 nm.

During a screen for β-lactams that could affect the cell cycle of *L*. *lactis*, we identified amoxicillin as a selective inhibitor of cell elongation (S12 Fig). *L*. *lactis* cells exposed to critical concentrations of amoxicillin (0.1 μg ml^-1^) were significantly rounder than untreated WT cells, with a length to width ratio of 1.21 ± 0.19 instead of 1.37 ± 0.17 (*t* test, *P* < 0.01, *n* = ~50) (Fig 8A). A lack of peripheral growth was confirmed when amoxicillin-treated cells were transferred onto methicillin-containing agar pads. In these conditions, cells started to swell instead of forming elongated filaments while when transferred to antibiotics-free medium, they continued to grow and recovered their typical ovoid shape after a few generations (S13 Fig). A similar swelling behavior was previously reported when *L*. *lactis pbp2b* mutant cells were treated with methicillin [9]. Thus, amoxicillin treatment selectively inhibits peripheral growth. However, the inhibition mechanism appears to be complex since *in vitro* PBP profiling in presence of a range of amoxicillin concentrations revealed that PBP1b, PBP2a, PBP2x, PBP2b, and DacA are potentially targeted by the antibiotic (data not shown).

Remarkably, amoxicillin-treated cells observed by TEM were nearly spherical but without mis-positioned septa, contrasting with TEM analysis of *pbp2b* mutant cells (compare Fig 8B and Fig 6). Moreover, there is no obvious differences in cell wall thickness between amoxicillin-treated and non-treated cells while it is highly heterogeneous in *pbp2b* mutant cells, and PG outgrowths (“piecrust”) were observed at future division sites as in the WT (Fig 6 and Fig 8B). These morphological structures were difficult to observe in *pbp2b* mutant cells (Fig 6 and data not shown).

Altogether, these results suggest that a fully active peripheral growth is not required for a correct positioning of the division machinery. This also indicates that the cell shape (ellipsoid *versus* spherical) does not seem to be a key determinant for the selection of the future division site in *L*. *lactis*.

## Discussion

### PBP2b localizes at the division site in both vegetative and filamentation cell cycles

*L*. *lactis* is an interesting ovococcal model regarding cell cycle since it displays a strict elongation phase before constriction (Fig 1) and it is capable to uncouple cell elongation and division in defined [9] or methicillin-induced conditions (Fig 3). Based on these unique specificities, we investigate the positioning of the Z ring and PBP2b using fluorescent reporter proteins during both vegetative and filamentation growth (Figs 2, 4 and 5).

**Fig 9:**
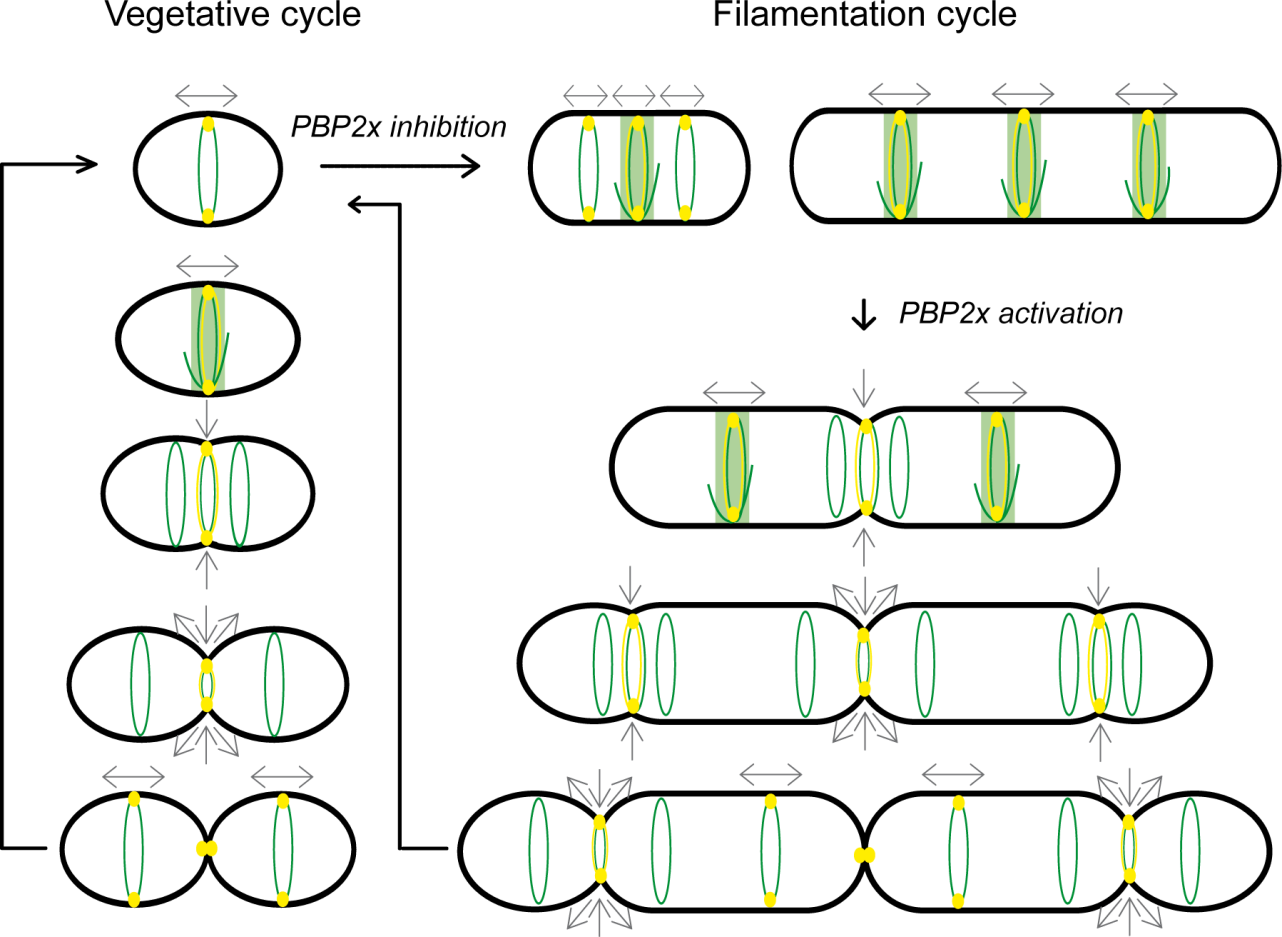
Model for FtsZ-directed dynamics of PBP2b during vegetative and filamentation cycles of *L*. *lactis*. FtsZ structures are shown in green. PBP2b is depicted as a yellow oval. Peripheral localization of the proteins is shown as a ring of the corresponding color. Direction of peripheral and septal growth is shown by grey arrows. The vegetative cell cycle (left) is divided in two separate phases as determined by FtsZ rings structural changes and spatio-temporal localization of PBPs, including PBP2b. During the elongation-only phase (two top cells), FtsZ equatorial ring exhibits a dynamic structure and the PBP2b-dependent peripheral growth mediates cell elongation at mid-cell. At the time of cell constriction, the Z ring segregates into 3 discrete rings; a central constricting ring directing cell division and two lateral rings that move apart as peripheral growth continues from PBP2b located at the septum. After completion of cell division, the elongation-specific PBP2b relocates to the new equatorial FtsZ rings of the newborn cells to reinitiate the cell cycle. The methicillin-induced filamentation cycle (right) results from PBP2x inhibition and reactivation following methicillin removal. During filamentation, PBP2b-dependent peripheral growth mediates cell elongation from a pre-septal position. During filament reversion, FtsZ-directed dynamics of PBP2b takes place as observed during the vegetative cycle but in a highly hierarchical manner starting from the center of the filament.

At the beginning of the vegetative cycle, the PBP2b-associated machinery joins the equatorial ring of FtsZ at the middle of the newborn cell (Fig 2B and 2C and Fig 9). During the 'elongation-only' phase, FtsZ rings appear to be highly dynamic and associated to the PBP2b-containing machinery, which forms parietal patches when examined by fluorescence microscopy (Fig 1B and Fig 2C). During this phase, FtsZ rings appear to split, generating 'V-like' structures or closely spaced rings prior to position at mid-cell for the next elongation/division cycle. Interestingly, similar structural changes of the FtsZ ring have been shown to take place during the pre-divisional stages of *S*. *pneumoniae* [43] and various rod-shaped bacteria [44–46]. Further understanding the dynamics of FtsZ ring during *L*. *Lactis* growth requires deeper investigations based on monocopy fluorescent protein expression and high-resolution microscopy.

In ovococcal cells, reorganization of FtsZ coupled to PG synthesis could be functionally analogous to the coupling between MreB and motion of the elongation machinery in rod-shaped bacteria [47,48]. Consistent with a dual role of FtsZ in cell elongation and cell division, depletion of FtsZ in *S*. *pneumoniae* led to cell swelling rather than to cell filamentation as is generally observed in rod-shaped bacteria [49]. In *L*. *lactis*, all PBPs, including the elongation-specific PBP2b, remain associated to the equatorial zone during most of the cell cycle (Fig 2B and 2C and Fig 9). This was also observed during filament formation and reversion where PBP2b appears to work from pre-existing (filament growth) or newly assembled (filament reduction) FtsZ rings to promote cell elongation (Fig 4, Fig 5, and Fig 9). These results are also in full agreement with our previous model of the filamentation cell-cycle in *L*. *lactis*, based on fluorescent vancomycin and FtsK-GFP localization patterns [9]. Altogether, the data converge towards a model where most of the peripheral growth is associated with the septal or pre-septal zone in *L*. *lactis* (Fig 9).

### The absence of PBP2b inhibits both peripheral growth and proper septation in *L*. *lactis*

As opposed to the well-established role of PBP2b in peripheral growth of ovococci [3,9,10], our finding that PBP2b also contributes to the positioning of the division machinery in *L*. *lactis* (Figs 6 and 7) was unexpected [10,50]. In addition, the inactivation of *pbp2b* is viable in *L*. *lactis* while it is lethal in *S*. *pneumoniae* [10,14]. Recently, the screening for PBP2b lethality suppressors identified the protein Spd1346 as being responsible for the lethal phenotype [51]. Spd1346 is a functional homologue of the endo-lytic transglycosylase MtlG of *E*. *coli*, which localizes with other peripheral PG synthesis proteins in *S*. *pneumoniae* [51]. It was hypothesized that the alteration of peripheral growth by PBP2b inactivation could lead to uncontrolled activation of MltG leading to cell death [51,52]. In addition, MltG implication in cell elongation of *S*. *pneumoniae* was confirmed by depletion experiments resulting in cells with increased sphericity [51]. The viability and the phenotype of *pbp2b* mutants in other ovococci were therefore questioned regarding the presence of suppressors of MltG activity [51]. *L*. *lactis* MG1363 (parental strain of NZ3900 used in this study) encodes one orthologous protein (Llmg0609) that displays 49% of identity with the conserved YceG domain of Spd1346. In order to verify the integrity of the *llmg0609* gene in the *L*. *lactis pbp2b* mutant, its locus was sequenced and no mutation/alteration could be detected (data not shown). A second suppressor of the lethality of PBP2b deficiency in *S*. *pneumoniae*, named EloR (Spr11851), was recently identified [52]. This protein, also present in *L*. *lactis* (Llmg0144), was proposed to be a regulator of cell elongation [52]. Consistently, its inactivation in *S*. *pneumoniae* led to shorter cells [52]. As these two suppressor mutations identified in *S*. *pneumoniae* altered the peripheral growth, the *pbp2b* mutant of *L*. *lactis* was reverted by allowing the excision of the disruption vector. The defects in cell elongation and septation were completely suppressed in the revertants (S10 Fig), showing that alterations of cell morphology is fully linked to the inactivation of the *pbp2b* locus and is not due to additional suppressor mutations localized elsewhere in the chromosome. Finally, complementation experiments with a plasmid-borne copy of *pbp2b* abrogated all morphological defects (Fig 7), confirming the absence of polar effects on adjacent genes due to *pbp2b* disruption. Altogether, these genetic data confirm that PBP2b has a bifunctional role in cell elongation and selection of the division site in *L*. *lactis*.

### What is the link between PBP2b and septation?

In *S*. *pneumoniae*, it was recently shown using a bacterial 2-hybrid system that PBP2b interacts with the PG transglycosylase RodA, MreD, and DivIVA, forming a complex involved in cell elongation [50]. However, the existence of separate complexes for elongation and division or the presence of a single 'dynamic' complex for both functions remains a matter of debate in ovococci [1,3,17,50]. Intriguingly, the PBP2b-depleted mutant of *L*. *lactis* exhibits strong defects in septum positioning and synthesis that could originate, either from the absence of the protein in the PG biosynthesis machinery *per se*, or from the lack of its specific transpeptidase activity. To investigate this aspect, we performed a complementation experiment with a catalytic mutant of PBP2b. This complementation was unable to restore the morphological defects regarding cell elongation and septum positioning (Fig 7 and S11 Fig), strongly suggesting a key contribution of the PBP2b transpeptidase activity to the observed phenotype. Another important aspect that could impact on the positioning of the division site is the cell shape itself. For instance, It is well established that DivIVA, which is involved in both division and elongation in *S*. *pneumoniae* [17,50,53], localizes to cell regions where the membrane has the strongest negative curvature, that is the septal region or the cell poles [53]. In this work, we identified amoxicillin as a specific inhibitor of peripheral growth (Fig 8 and S12 and S13 Figs). Interestingly, the resulting round cells were not affected in septum positioning, suggesting that altered cell shape by itself is not a key determinant for the selection of the division site in *L*. *lactis*. Based on the septal localization of PBP2b and the importance of its catalytic activity in the default of septum positioning, it is tempting to propose that PBP2b could contribute to the synthesis of a PG with a specific composition at the division site. In *S*. *pneumoniae*, a specific PG structure (i.e. “piecrust”) or a PG with a different composition at the constricting septum/future division site has been hypothesized to be required for the specific positioning of the PG-binding protein MapZ (also present in *L*. *lactis*, Llmg0772), which in turn recruits FtsZ to start the next division cycle [18,19,54]. Intriguingly, we were not able to visualize PG outgrowths (“piecrust”) in PBP2b-deficient cells while they are easily detected in wild-type (Fig 6) or amoxicillin-treated cells (Fig 7B). Consistent with the idea of a PBP2b-mediated specific PG assembly, the PG composition of PBP2b-depleted cells of *S*. *pneumoniae* is altered with fewer directly linked and more branched stem peptides, suggesting that PBP2b has a preferential transpeptidase activity for unbranched PG precursors in this species [50,55]. A preliminary analysis of the PG composition of the *L*. *lactis pbp2b* mutant shows an increased ratio of tetra- and tri-peptides *versus* penta-peptides, indicating either a higher activity of D,D- and D,L-carboxypeptidases (i.e. DacA and DacB, respectively) on pentapeptides or a lower incorporation of processed peptides due to PBP2b inactivation (unpublished data). Remarkably, a deficiency in D,D-carboxypeptidase activity in *L*. *lactis* (DacA^-^) and *S*. *pneumoniae* (PBP3^-^) resulted in similar defects as observed here for the *L*. *lactis pbp2b* mutant [12,39–41]. In those mutants, peripheral growth is inhibited [12,39–41], PG outgrowths (piecrusts) could not be visualized [41], and septa are misplaced [12,41]. In *L*. *lactis*, it seems that the transpeptidase PBP2b and the D,D-carboxypeptidase DacA are functionally interconnected for allowing proper peripheral growth and correct positioning of the division site. The molecular details of this interconnection requires further investigations.

## Supporting information

S1 Fig Expression and stability of the Venus fusion proteins. (PDF)

S2 Fig Time-lapse imaging of FtsZ during the vegetative cell cycle of *L. lactis* (additional example). (PDF)

S3 Fig Time-lapse fluorescence profiles of FtsZ during the vegetative cell cycle of *L. lactis*. (PDF)

S4 Fig Fluorescence profiles of Ve-PBP2b during the vegetative cell cycle of *L. lactis*. (PDF)

S5 Fig Distribution of FtsZ and PBP2b rings according to filament length. (PDF)

S6 Fig Localization of PBPs with respect to FtsZ in methicillin-induced filaments. (PDF)

S7 Fig Time-lapse imaging of FtsZ during filament reversion (additional example). (PDF)

S8 Fig Time-Lapse fluorescence profiles of FtsZ-Ve and Ve-PBP2b during filament reversion. (PDF)

S9 Fig Growth defect of the *pbp2b* mutant. (PDF)

S10 Fig Cell length and width of WT compared to revertants of the *pbp2b* mutant. (PDF)

S11 Fig Complementation assay of the *pbp2b* mutant with the catalytic mutant of PBP2b (PBP2b*) in presence of nisin 0.1 ng ml^-1^. (PDF)

S12 Fig Screen for β-lactams affecting cell elongation or division in *L. lactis*. (PDF)

S13 Fig Swelling of amoxicillin pre-treated WT cells in presence methicillin. (PDF)

S1 Table Primers used for cloning and validation. (PDF)

S1 Movie Time lapse imaging of FtsZ-Venus during cell growth. Phase-contrast microscopy.
(AVI)

S2 Movie Time lapse imaging of FtsZ-Venus during cell growth. Fluorescence microscopy.
(AVI)

S3 Movie Time lapse imaging of FtsZ-Venus during cell growth. Phase-contrast microscopy (additional example).
(AVI)

S4 Movie Time lapse imaging of FtsZ-Venus during cell growth- Fluorescence microscopy (additional example). (AVI)

S5 Movie Time-lapse of the reversion of methicillin induced filaments expressing FtsZ-Venus. Phase-contrast microscopy.
(AVI)

S6 Movie Time-lapse of the reversion of methicillin induced filaments expressing FtsZ-Venus. Fluorescence microscopy.
(AVI)

S7 Movie Time-lapse of the reversion of methicillin induced filaments expressing FtsZ-Venus. Phase-contrast microscopy (additional example).
(AVI)

S8 Movie Time-lapse of the reversion of methicillin induced filaments expressing FtsZ-Venus. Fluorescence microscopy (additional example).
(AVI)

S9 Movie Time-lapse of the reversion of methicillin induced filaments expressing Venus-PBP2b. Phase-contrast microscopy.
(AVI)

S10 Movie Time-lapse of the reversion of methicillin induced filaments expressing Venus-PBP2b. Fluorescence microscopy.
(AVI)
