# Supplementary material for: PBP2b plays a key role in both peripheral growth and septum positioning in Lactococcus lactis
Source: PLoS One. 2018 May 23;13(5):e0198014. doi: 10.1371/journal.pone.0198014 (PMC5965867; doi:10.1371/journal.pone.0198014)
Supplement: S1 Fig — (PDF) [file pone.0198014.s001.pdf]

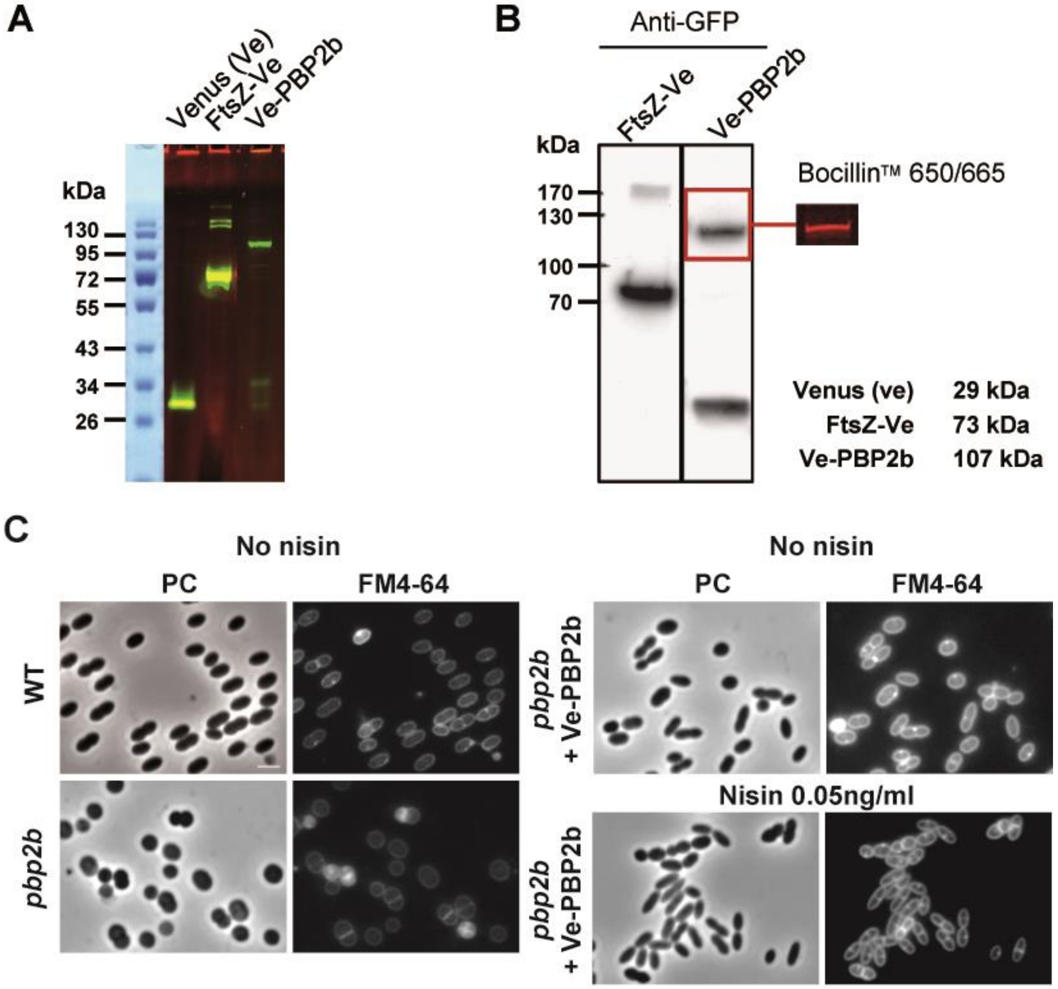

S1 Fig. Expression and stability of the Venus fusion proteins. (A) Total (Ve and FtsZ-Ve) and membrane (Ve-PBP2b) extracts from cells expressing Venus (Ve, 29 kDa), FtsZ-Venus (FtsZ-Ve, 73 kDa) and Venus-PBP2b (Ve-PBP2b, 107 kDa) proteins were separated on a pre-cast 4-20 % SDS-polyacrylamide gel. The gel was washed to remove SDS and visualized using a fluorescence scanner. (B) Western blotting of extracts expressing FtsZ-Ve and Ve-PBP2b using a specific anti-GFP antibody. Right inset, substrate-binding activity of Venus-PBP2b by staining the gel with Bocillin^{TM} 650/665 prior to florescence scanning. Molecular weight of size markers are shown on the left of the gels. (C) Complementation of *pbp2b* mutant with Venus-PBP2b fusion (Ve-PBP2b). Images of wild type (WT, NZ3900), *pbp2b* mutant, and *pbp2b* + Ve-PBP2b cells obtained by phase contrast (PC) and epifluorescence (membrane staining with FM4-64) microscopy. Cells were grown without (no nisin) or with nisin (0.05 ng ml^{-1}) as inducer of the expression of *venus::pbp2b* gene fusion. The recovery of the elongated cell shape in *pbp2b* + Ve-PBP2b cells shows the functionality of Ve-PBP2b.
