# Supplementary material for: PBP2b plays a key role in both peripheral growth and septum positioning in Lactococcus lactis
Source: PLoS One. 2018 May 23;13(5):e0198014. doi: 10.1371/journal.pone.0198014 (PMC5965867; doi:10.1371/journal.pone.0198014)
Supplement: S2 Fig — (PDF) [file pone.0198014.s002.pdf]

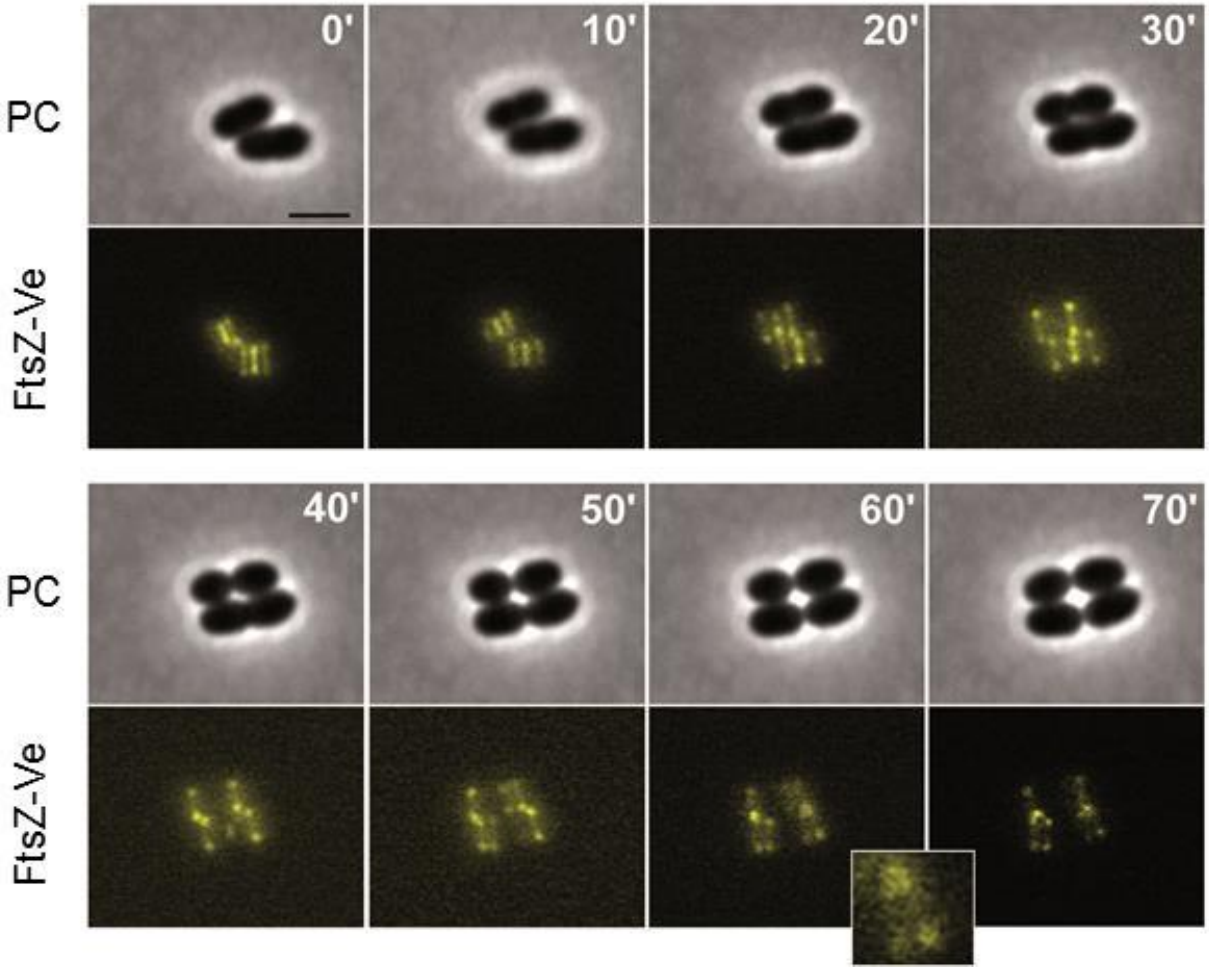

S2 Fig. Time-lapse imaging of FtsZ during the vegetative cell cycle of *L. lactis* (additional example). *L. lactis* cells expressing the FtsZ-Venus fluorescent protein were grown on agar pads and visualized by phase contrast (PC) and epifluorescence (FtsZ-Ve) microscopy (see S3-4 Movies). Pictures were taken every 10 min. The inset shows an enlargement of specific 'V-like' FtsZ structures frequently observed at the early stages of cell elongation. Scale bar, 2 µm.
