# Supplementary material for: PBP2b plays a key role in both peripheral growth and septum positioning in Lactococcus lactis
Source: PLoS One. 2018 May 23;13(5):e0198014. doi: 10.1371/journal.pone.0198014 (PMC5965867; doi:10.1371/journal.pone.0198014)
Supplement: S3 Fig — (PDF) [file pone.0198014.s003.pdf]

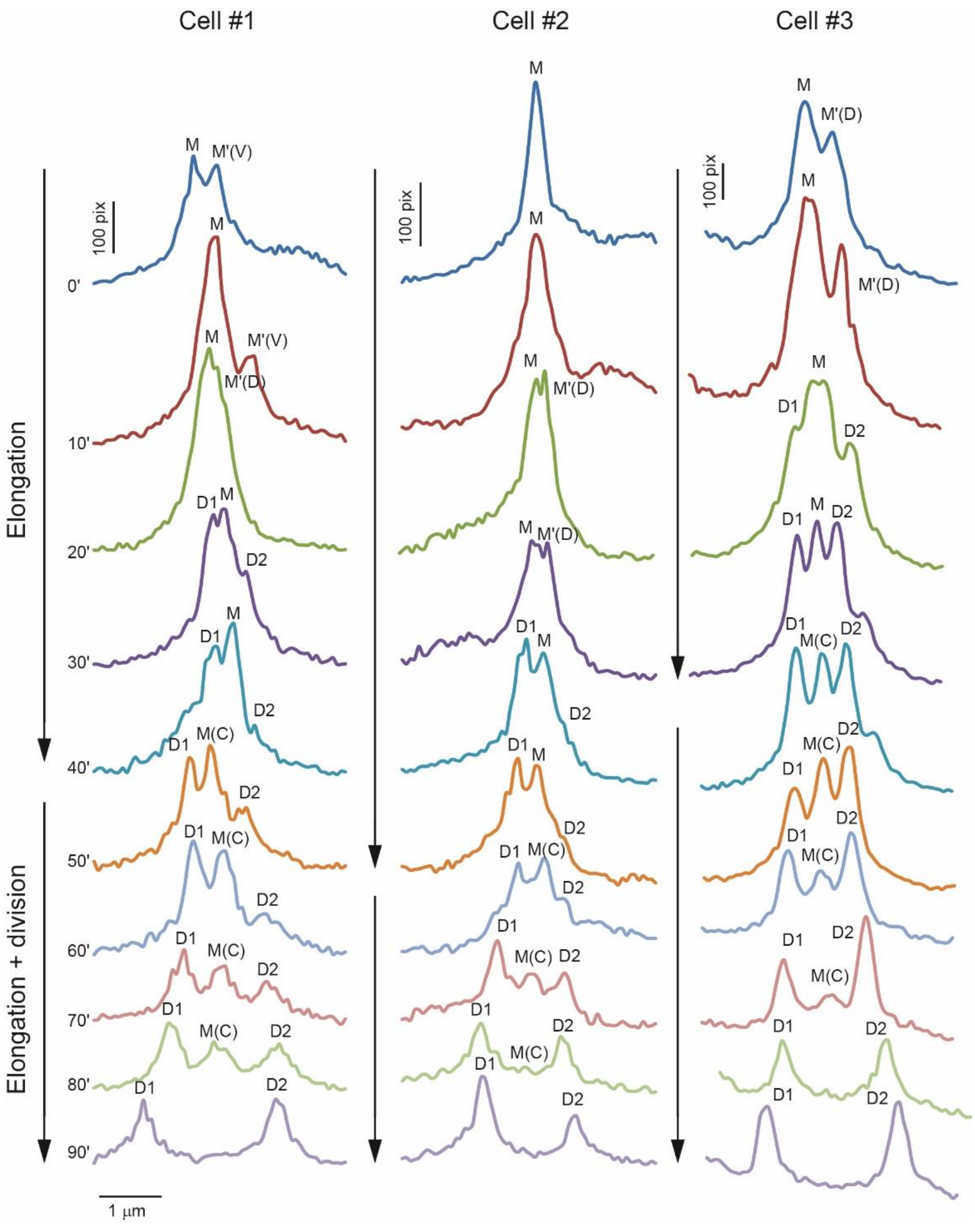

S3 Fig. Time-lapse fluorescence profiles of FtsZ during the vegetative cell cycle of *L. lactis*. Fluorescence profiles of FtsZ-Ve localization during the cell cycle of 3 representative cells (Cell #1, #2, and #3, strain NZ3900 [pGIBLD031]) collected from 3 independent time-lapse experiments. For cell #1 (shown in Fig. 1B), a median "V-shape" ring (M + M'(V)) and double rings (M + M'(D)), followed by the appearance of 2 lateral rings (daughter rings D1 and D2), are observed at the pre-divisional stage. For cells #2 and #3, only double rings (M + M'(D)) are observed at this stage. After the start of the constriction of the median ring (M(C)), distribution of lateral rings (D1 and D2) is similar between the three cells. Profiles were extracted every 10 min. Fluorescence scale, 100 pixels (pix). Microscopy images were analyzed with AxioVision.
