# Supplementary material for: PBP2b plays a key role in both peripheral growth and septum positioning in Lactococcus lactis
Source: PLoS One. 2018 May 23;13(5):e0198014. doi: 10.1371/journal.pone.0198014 (PMC5965867; doi:10.1371/journal.pone.0198014)
Supplement: S4 Fig — (PDF) [file pone.0198014.s004.pdf]

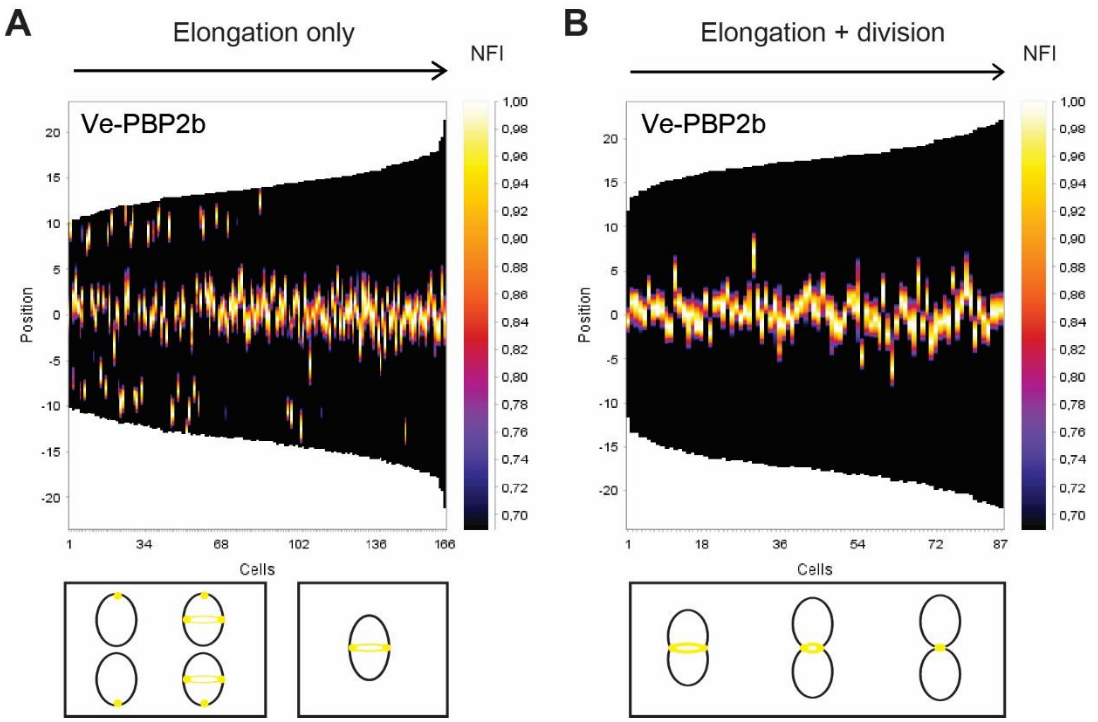

S4 Fig. Fluorescence profiles of Ve-PBP2b during the vegetative cell cycle of *L. lactis*. Normalized fluorescence pattern of cells (strain NZ3900 [pGIBLD041]) at pre-divisional stages (absence of constriction, *n* = 166) (A) and division stage (presence of constriction, *n* = 87) (B). The relative position from the center of the longitudinal axis (position 0) is in arbitrary units. The scale of normalized fluorescence intensity (NFI) is shown on the right of each panel. The schematic representation of the different patterns of Ve-PBP2b localization (yellow) is shown below each panel. This population analysis was performed from an independent experiment than shown in Fig. 2. Microscopy images were analyzed with MicrobeJ.
