# Supplementary material for: PBP2b plays a key role in both peripheral growth and septum positioning in Lactococcus lactis
Source: PLoS One. 2018 May 23;13(5):e0198014. doi: 10.1371/journal.pone.0198014 (PMC5965867; doi:10.1371/journal.pone.0198014)
Supplement: S5 Fig — (PDF) [file pone.0198014.s005.pdf]

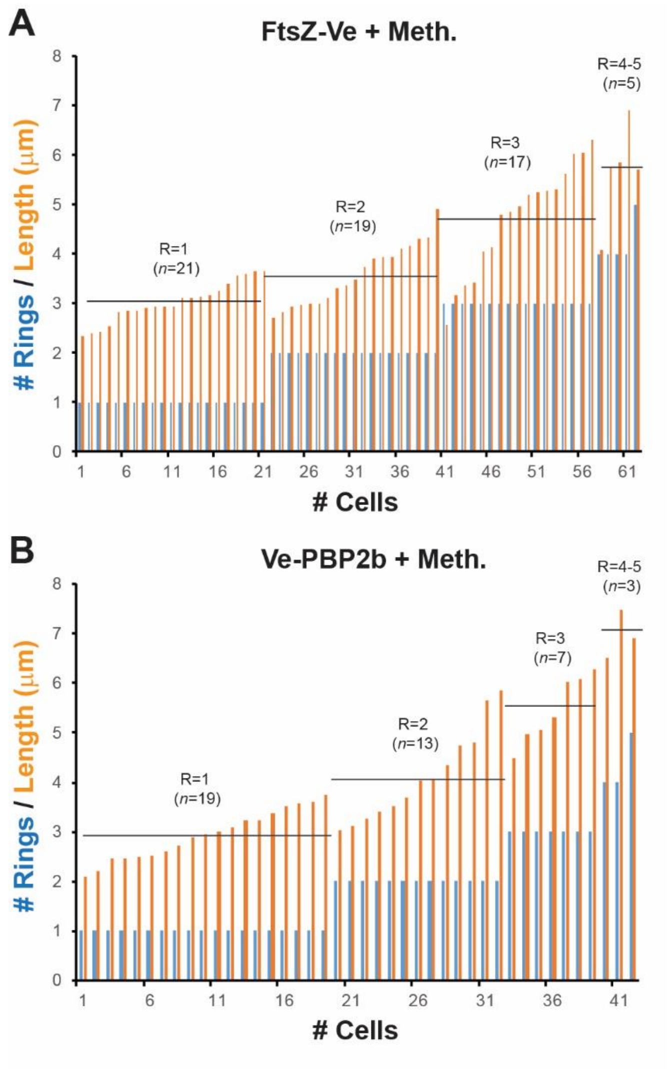

S5 Fig. Distribution of FtsZ and PBP2b rings according to filament length. *L. lactis* cells expressing FtsZ-Ve (strain NZ3900 [pGIBLD031]) (A) or Ve-PBP2b (strain NZ3900 [pGIBLD041]) (B) were grown in the presence of methicillin 1 µg ml^{-1} to induce filament formation and visualized by optical microscopy. Length (μm, orange bars) and ring number (R=1 to 5, blue bars) of filaments (*n* = 62 and 42, respectively) were measured from phase contrast and fluorescence images, respectively. Black bar, mean length value for each filament subpopulation separated by increased ring numbers. Microscopy images were analyzed with AxioVision.
