# Supplementary material for: PBP2b plays a key role in both peripheral growth and septum positioning in Lactococcus lactis
Source: PLoS One. 2018 May 23;13(5):e0198014. doi: 10.1371/journal.pone.0198014 (PMC5965867; doi:10.1371/journal.pone.0198014)
Supplement: S6 Fig — (PDF) [file pone.0198014.s006.pdf]

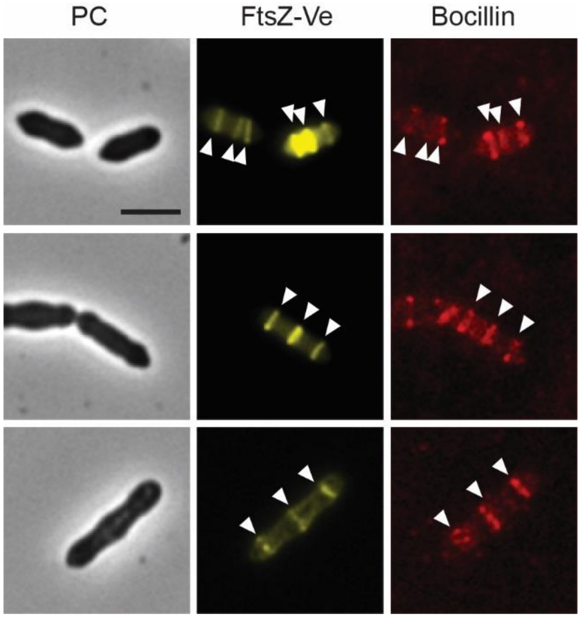

S6 Fig. Localization of PBPs with respect to FtsZ in methicillin-induced filaments. Filaments expressing FtsZ-Ve (strain NZ3900 [pGIBLD031]) were stained with Bocillin™650/665 and visualized by phase contrast (PC) and epifluorescence (FtsZ-Ve and Bocillin) microscopy. White arrows indicate bands of FtsZ-Ve/PBP co-localization.
