# Supplementary material for: PBP2b plays a key role in both peripheral growth and septum positioning in Lactococcus lactis
Source: PLoS One. 2018 May 23;13(5):e0198014. doi: 10.1371/journal.pone.0198014 (PMC5965867; doi:10.1371/journal.pone.0198014)
Supplement: S7 Fig — (PDF) [file pone.0198014.s007.pdf]

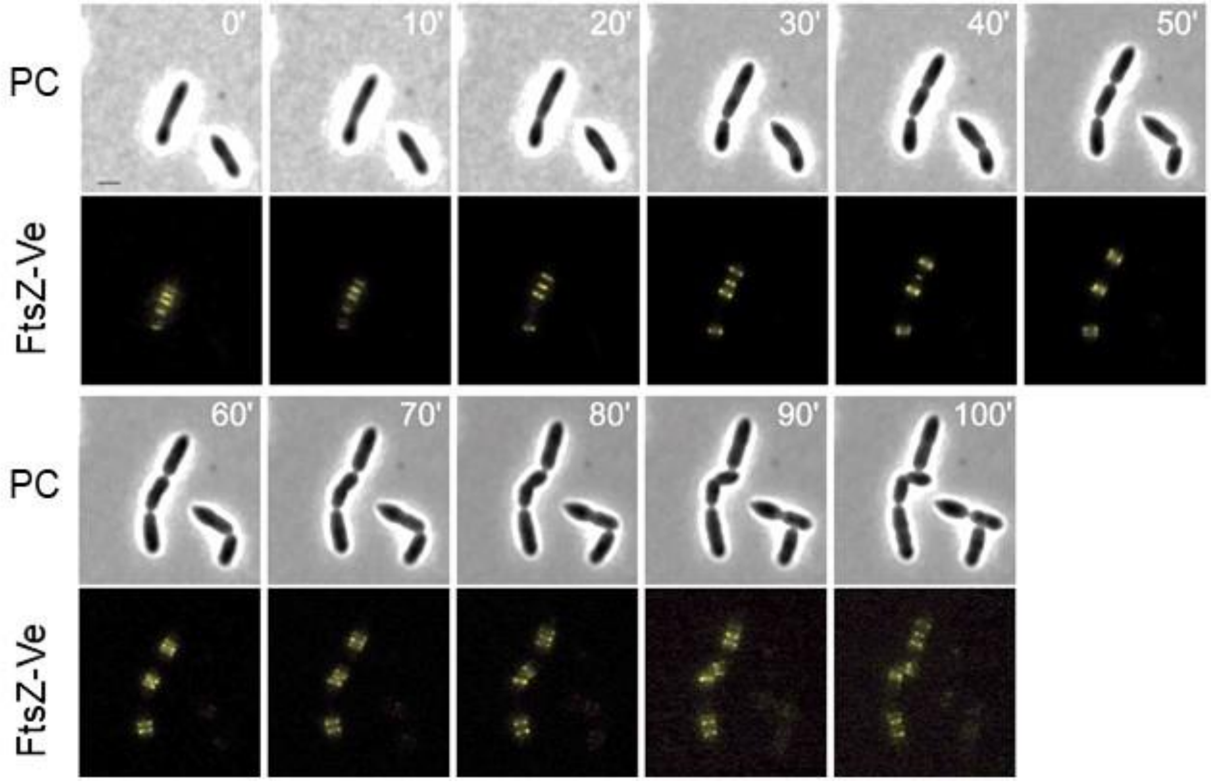

S7 Fig. Time-lapse imaging of FtsZ during filament reversion (additional example). *L. lactis* filaments expressing FtsZ-Ve (strain NZ3900 [pGIBLD031]) were grown on methicillin-free agar pads to reverse filamentation. The dynamics of FtsZ-Ve rearrangements was analyzed by time lapse imaging (see also S7-8 Movies). Top and bottom rows correspond to phase contrast (PC) and epifluorescence (FtsZ-Ve) microscopy, respectively. Pictures were taken every 10 min. Scale bars, 2 µm.
