# Supplementary material for: PBP2b plays a key role in both peripheral growth and septum positioning in Lactococcus lactis
Source: PLoS One. 2018 May 23;13(5):e0198014. doi: 10.1371/journal.pone.0198014 (PMC5965867; doi:10.1371/journal.pone.0198014)
Supplement: S8 Fig — (PDF) [file pone.0198014.s008.pdf]

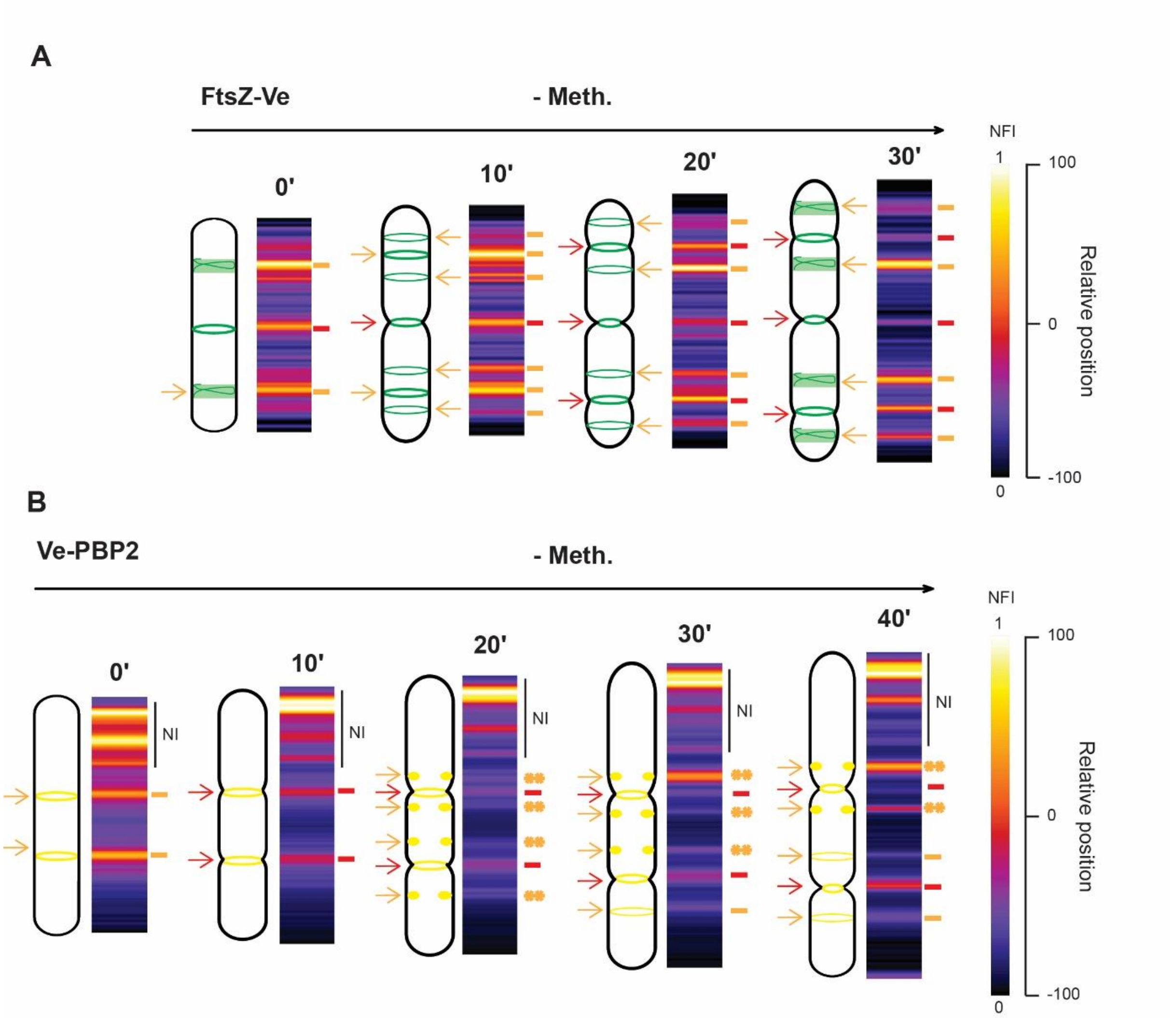

S8 Fig. Time-Lapse fluorescence profiles of FtsZ-Ve and Ve-PBP2b during filament reversion. (A) Normalized fluorescence pattern of cells expressing FtsZ-Ve (strain NZ3900 [pGIBLD031]) (A) and Ve-PBP2b (strain NZ3900 [pGIBLD041]) (B) during filament reversion (removal of methicillin, -Meth.). Analyzed filaments are those shown in Fig. 5. Profiles were extracted every 10 min. The relative position from the center of the longitudinal axis (position 0) is in arbitrary units. The scale of normalized fluorescence intensity (NFI) is shown on the right of each panel. The schematic representation of the different patterns of localization of FtsZ-Ve (green) or Ve-PBP2b (yellow) is shown on the left of each fluorescence pattern. Localization of fluorescent rings at the constricting septum and future division sites is highlighted with red and orange arrows/bars, respectively. **, symmetric foci. NI, not interpreted due to the misshaping of the filament at this position. Microscopy images were analyzed with MicrobeJ.
