# Supplementary material for: PBP2b plays a key role in both peripheral growth and septum positioning in Lactococcus lactis
Source: PLoS One. 2018 May 23;13(5):e0198014. doi: 10.1371/journal.pone.0198014 (PMC5965867; doi:10.1371/journal.pone.0198014)
Supplement: S9 Fig — (PDF) [file pone.0198014.s009.pdf]

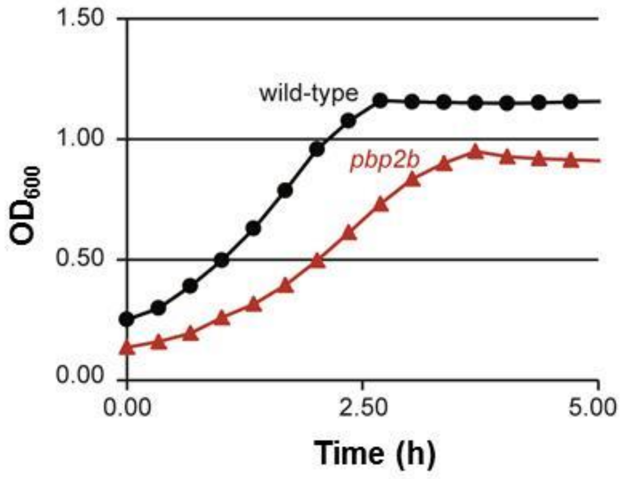

S9 Fig. Growth defect of the *pbp2b* mutant. Growth curves of wild-type and *pbp2b* mutant cells were grown in M17G supplemented with 5µg ml^{-1} of erythromycin when appropriate. For each strain, the OD_{600} was monitored every 20 min. The growth curves presented are representative of various independent experiments.
