# Supplementary material for: PBP2b plays a key role in both peripheral growth and septum positioning in Lactococcus lactis
Source: PLoS One. 2018 May 23;13(5):e0198014. doi: 10.1371/journal.pone.0198014 (PMC5965867; doi:10.1371/journal.pone.0198014)
Supplement: S10 Fig — (PDF) [file pone.0198014.s010.pdf]

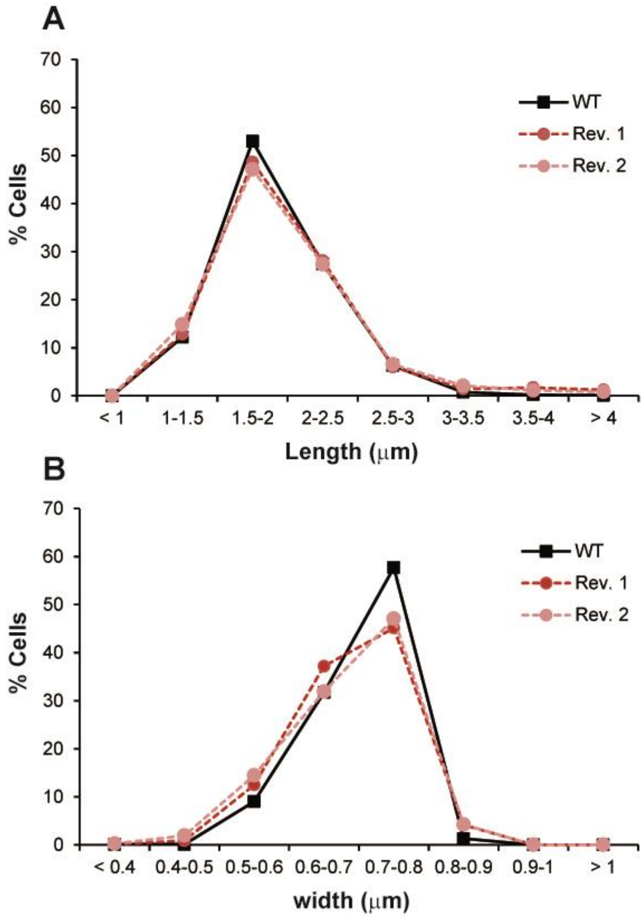

S10 Fig. Cell length and width of WT compared to revertants of the *pbp2b* mutant. The *pbp2b* mutant was grown in M17G without antibiotics during 20 generations. Two erythromycin sensitive clones (rev. 1 and rev. 2) resulting from excision of the disruptive vector from the *pbp2b* locus were selected. Cell length and width were measured from microscopy images using MicrobeTracker (*n* > 50 cells).
