# Supplementary material for: PBP2b plays a key role in both peripheral growth and septum positioning in Lactococcus lactis
Source: PLoS One. 2018 May 23;13(5):e0198014. doi: 10.1371/journal.pone.0198014 (PMC5965867; doi:10.1371/journal.pone.0198014)
Supplement: S11 Fig — (PDF) [file pone.0198014.s011.pdf]

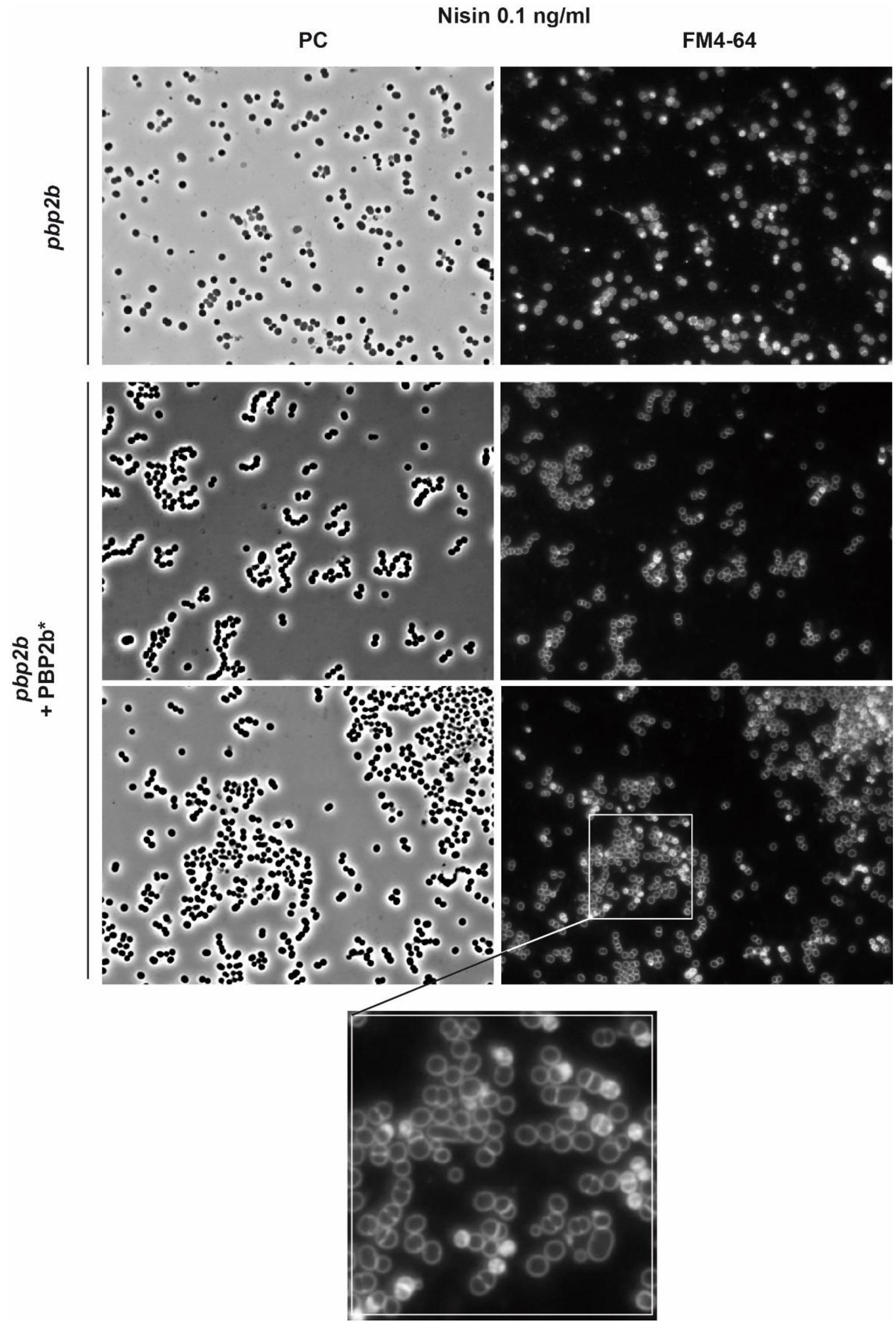

S11 Fig. Complementation assay of the *pbp2b* mutant with the catalytic mutant of PBP2b (PBP2b*) in presence of nisin 0.1 ng ml^{-1}. Images of *pbp2b* mutant and *pbp2b* + PBP2b* cells obtained by phase contrast (PC) and epifluorescence (membrane staining with FM4-64) microscopy.
