# Supplementary material for: PBP2b plays a key role in both peripheral growth and septum positioning in Lactococcus lactis
Source: PLoS One. 2018 May 23;13(5):e0198014. doi: 10.1371/journal.pone.0198014 (PMC5965867; doi:10.1371/journal.pone.0198014)
Supplement: S12 Fig — (PDF) [file pone.0198014.s012.pdf]

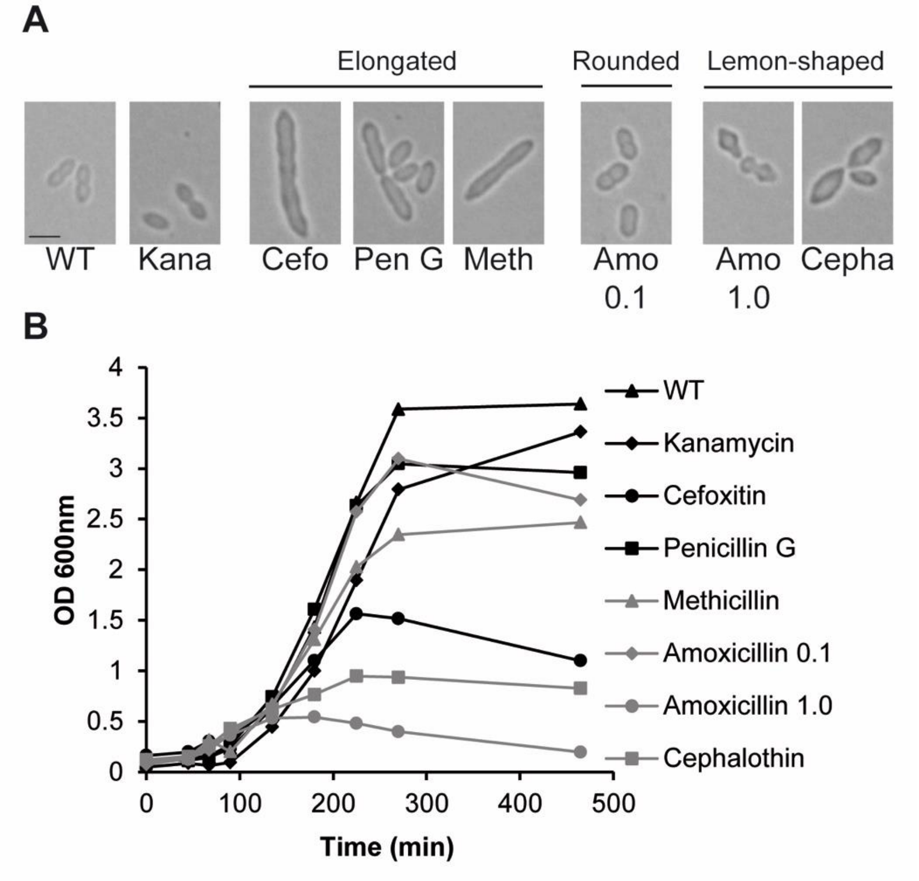

S12 Fig. Screen for β-lactams affecting cell elongation or division in *L. lactis*. (A) Micrographs of NZ3900 cells untreated (WT) or treated with 12 µg ml^{-1} of kanamycin (Kana, control), 64 µg ml^{-1} of cefoxitin (Cefo), 0.1 µg ml^{-1} of penicillin G (Pen G), 1 µg ml^{-1} of methicillin (Meth), 0.1 µg ml^{-1} of amoxicillin (Amo 0.1), 1 µg ml^{-1} of amoxicillin (Amo 1.0), and 10 µg ml^{-1} of cephalosporin C (Cepha). Cells were collected in early exponential growth and observed in bright field microscopy. Scale bar: 2µm. (B) Growth curves of NZ3900 cells untreated or treated with antibiotics as reported in panel A.
