# Supplementary material for: PBP2b plays a key role in both peripheral growth and septum positioning in Lactococcus lactis
Source: PLoS One. 2018 May 23;13(5):e0198014. doi: 10.1371/journal.pone.0198014 (PMC5965867; doi:10.1371/journal.pone.0198014)
Supplement: S13 Fig — (PDF) [file pone.0198014.s013.pdf]

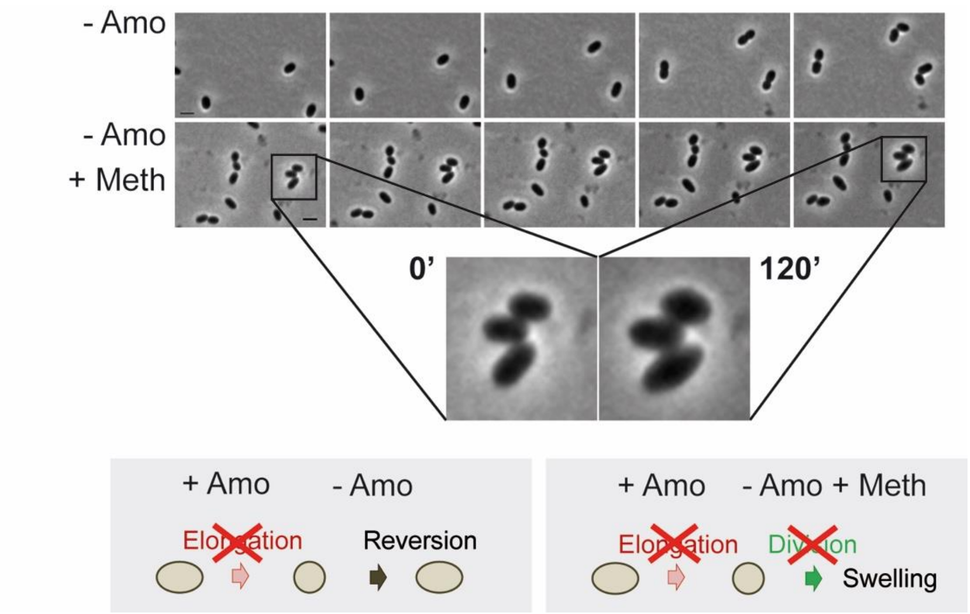

S13 Fig. Swelling of amoxicillin pre-treated WT cells in presence methicillin. Amoxicillin pre-treated cells were grown on fresh agar pads devoid of amoxicillin (-Amo) supplemented with 1 µg ml^{-1} of methicillin (+ Meth) or not as indicated. - Amo, reversion of the inhibition of cell elongation; -Amo + Meth, absence of reversion and cell swelling (see insets) Cells were visualized by phase contrast microscopy and photographed every 5 min for 120 min. Only the pictures taken with 30 min intervals are shown. Scale bars, 2 µm.
