# Supplementary material for: PBP2b plays a key role in both peripheral growth and septum positioning in Lactococcus lactis
Source: PLoS One. 2018 May 23;13(5):e0198014. doi: 10.1371/journal.pone.0198014 (PMC5965867; doi:10.1371/journal.pone.0198014)
Supplement: S1 Table — (PDF) [file pone.0198014.s014.pdf]

S1 Table. Primers used for cloning and validation.

Primer	Sequence (5' to 3')	Mutant strain or plasmid
**Primers used for the validation of *pbp* disruption**		
BlD-PBP1aUpNcoI	aaccATGgTGGTTAATGCTG	BLD001
BlD-PBP1aDownSacI	agagctcATTTAGCCACCATTAGC	
BlD-PBP1bUpNcoI	aaccATGgAAAAAGACTCAATTG	BLD002
BlD-PBP1bDownSacI	agagctcTTAGTTACTTTTTGATGAGG	
BlD-PBP2aUpPciI	aaaaaaacATGtCTGAAAATAAG	BLD003
BlD-PBP2aDownSacI	agagctcTTAGTTGCCAAAAACC	
BlD-PBP2bUpNcoI	aaccATGgCAGTTAATAAAAG	BLD004
BlD-PBP2bDownXbaI	atctagaTTAATTTGTTCGAAAAGC	
BlD-DacAUpNcoI	aaccATGgAAAAAATTGCAATTATTTTTTG	BLD006
BlD-DacADownSacI	agagctcTTAGAGTTTTTCATTAAC	
**Primers used for the construction of expression vectors**		
BlD-PBP2bUpNcoI	aaccATGgCAGTTAATAAAAG	pGIBLD027
BlD-PBP2bDownXbaI	atctagaTTAATTTGTTCGAAAAGC	
PBP2B*Up	AGGGgCTGTTGTGAAAATGGGAACATTG	pGIBLD0271
PBP2B*Down	CCATTTTCACAACAGcCCCTGGCGTAAAGAC	
MBO-FtsZUp4X4	ATATACATGTGGCCATGGAATTTTCATTTGATACAGACTTA	pGIBLD031
MBO-FtsZDown4X4	ATCTAGATAGATTAATTTTACGGAAGAATGGTGG	
BlD-PBP2bUp4X4	ATATACATGTGGCCATGGCAGTTAATAAAAGAAAAAAC	pGIBLD041
BlD-PBP2bDown4X4	ATCTAGAATTTGTTCGAAAAGCTTCGTT	
**Primers used for the validation of the acquisition of expression vectors**		
MBO-4X4UpOl1	TTTGTTAGATACAATGATTTCG	pNZ8048 and derivatives
MBO-4X4DownOl2	TCAAGCCTTGGTTTTCTAATTTTGG	

[a] Underlined sequences correspond to restriction enzymes sites.
